# From Eyes to Cameras to Kinetics: Computer Vision for Chemical Process Monitoring

**DOI:** 10.1021/acs.accounts.6c00137

**Published:** 2026-05-14

**Authors:** Henry Barrington, Kristin Donnachie, Calum Fyfe, Timothy J. D. McCabe, Cameron Ward, Marc Reid

**Affiliations:** Department of Pure and Applied Chemistry, 3527 University of Strathclyde, Glasgow G1 1XW, U.K.

## Abstract

Chemical manufacturing is dominated by color changes that offer quantifiable insights into a huge variety of transformations. Yet, despite the vast array of chemical phenomena we experience by eye, few reaction monitoring tools are designed to measure what we see. This Account describes our journey developing computer vision to turn any camera into a chemistry-agnostic reaction monitoring tool able to transform everyday visual observations into quantitative kinetic data. Chemists routinely record color changes, precipitation, and mixing patterns without systematically leveraging this information. Our work emerged from an idea inspired by early career resource constraints: if visual changes can be described qualitatively, they can be quantified digitally, and cheaply. This insight, catalyzed by Raspberry Pi camera experiments and a serendipitous industrial need, led us to develop computer vision methods that enable bulk process monitoring across scales. Central to our contributions is the development of *Kineticolor*, a software platform converting video recordings into time-resolved kinetic information. Beyond color averaging, we developed spatially resolved analyses, providing time-resolved texture metrics to reveal mixing phenomena, even in small scale applications where the temptation is often to ignore mixing effects. Crucially, our methods are scale-agnostic, meaning identical algorithms apply from microscale wells to industrial reactors. Our industry-focused collaborations have highlighted computer vision’s complementary role in process monitoring: while molecular techniques provide chemical identity, noninvasive visual methods capture bulk phenomena missed by point measurements. Our work serves modern automation laboratories looking for their next complementary data stream, as well as enabling accessible monitoring using standard cameras for resource-constrained environments. If chemists observe visual changes in laboratory notebooks, computer vision can quantify those tacit insights. The principle is simple: if you can *see* it, these methods can *measure* it.

## Graphical abstract

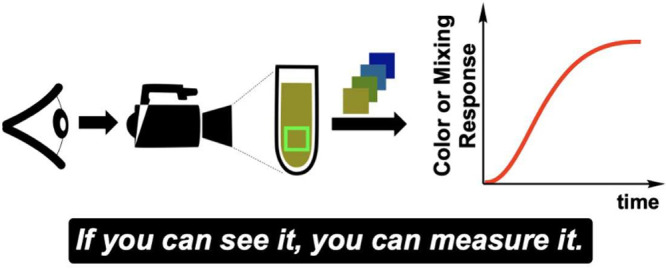

## Key References

- Barrington, H.; McCabe, T. J. D.; Donnachie, K.; Fyfe, C.; McFall, A.; Gladkikh, M.; McGuire, J.; Yan, C.; Reid, M. Parallel and High Throughput Reaction Monitoring with Computer Vision. *Angew. Chem. Int. Ed.* **2025**, *64*, e202413395.^8^ Kinetics from reactions on well plates or side-by-side.
- Yan, C.; Cowie, M.; Howcutt, C.; Wheelhouse, K. M. P.; Hodnett, N. S.; Kollie, M.; Gildea, M.; Goodfellow, M. H.; Reid, M. Computer vision for noninvasive monitoring of catalyst degradation and product formation kinetics. *Chem. Sci.* **2023**, *14*, 5323–5331.^5^ Kinetics of palladium catalyst lifetime.
- Yan, C.; Fyfe, C.; Minty, L.; Barrington, H.; Jamieson, C.; Reid, M. Computer vision as a new paradigm for monitoring of solution and solid phase peptide synthesis. *Chem. Sci.* **2023**, *14*, 11872–11880.^6^ Camera-enabled monitoring of challenging multiphase systems.
- Barrington, H.; Dickinson, A.; McGuire, J.; Yan, C.; Reid, M. Computer Vision for Kinetic Analysis of Lab- and Process-Scale Mixing Phenomena. *Org. Proc. Res. Dev.* **2022**, *26*, 3073–3088.^4^ Bridging tools for chemists and chemical engineers for understanding mixing effects in reaction design.

## 1 Introduction

Color changes are ubiquitous in chemistry. A palladium-catalyzed coupling slowly transitions from pale yellow to deep amber as the catalyst ages and solids form.^1^ An organic synthesis mixture shifts through a spectrum of hues, perhaps from colorless to vibrant orange to deep purple, or from a light to a subtly darker yellow, each shade reflecting reaction progress, side reactions, and molecular transformations occurring within. From crystallization turbidity to color-changing drug tests, visual observations of reaction bulk contain kinetic information we rarely quantify. This highlights an underexploited opportunity: we are visual scientists whose notebooks contain qualitative observations (“solution turned dark”, “precipitate formed”) that we rarely turn into data. If we did, new mechanistic insights, process optimization strategies, and discovery efforts would be accelerated.

Our wealth of untapped visual information becomes even more notable when we consider its accessibility. Unlike complementary analytical techniques that require specialized equipment, sample preparation, or invasive sampling procedures, visual monitoring is inherently noninvasive, universally available, and relatively inexpensive.^2,3^ Bridging what we see and what we can measure requires computational tools to transform visual observations into quantitative kinetic data. It is this gap between the richness of visual phenomena in chemistry and our systematic quantification of these observations that our *Kineticolor* research team set out to address through the development of computer vision approaches to reaction monitoring.^4–8^

### 1.1 Our Beginnings in Computer Vision

Our computer vision pursuit began from resource constraints. Early career projects required reaction monitoring methods beyond our budget. A Raspberry Pi kit provided an opportunity to explore converting visual observations into quantitative data.^9^ Among the circuit boards, breadboards, wires, and widgets was a small camera module that, once connected, revealed a straightforward means through which to turn videos of a red dye diffusing through transparent liquid into a numerical picture of that recording.

### 1.2 Computer Vision for Analytical Chemistry (CVAC)

The field of computer vision for analytical chemistry (CVAC) grew steadily over the first two decades of the 21st century.^10–14^ While the field itself is too vast to meaningfully review under the single umbrella of “computer vision”, we have been inspired by a number of key contributions to time-resolved computer vision, before and during our development of our *Kineticolor* software.^15^

From visualizing microwave chemistry^16^ to the quantification of chocolate bloom^17^ and the effects of fish grilling,^18^ the first signal of opportunity came from understanding the wide variety of reported applications that showed the benefits from analyzing images and video frames to quantify process evolution (Figure 1). Early work by Tanguy et al. in 2007 demonstrated the power of colorimetric image analysis for mixing time determination in stirred reactors, circumventing the subjectivity inherent in visual assessment by analyzing color distributions across thousands of image pixels.^19^ Far from the world of liquid–liquid mixing, related imaging approaches have been shown to quantify the homogenization of two food powders.^20^ In metallurgy, Alberto et al. used digital images to quantify color evolution related to the laser-induced chemical changes in iron oxide pigments.^21^ The conceptual leap toward broader application in synthetic chemistry was articulated in Ley’s influential 2013 review, which envisioned cameras as transformative tools for organic synthesis monitoring.^22^

**Figure 1:**
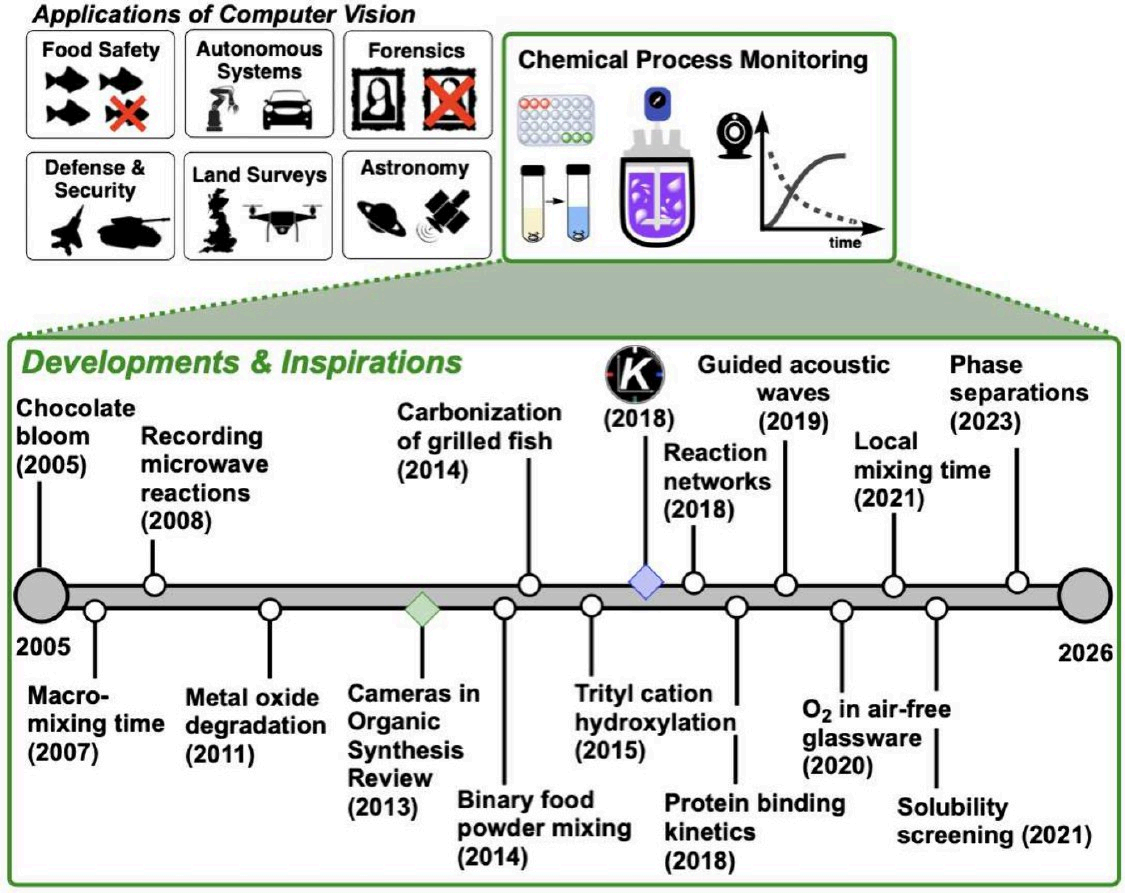
Toward time-resolved computer vision as a process monitoring tool in chemistry. The top panel positions chemical process monitoring among broader computer vision applications, while the timeline below highlights key inspirations from the literature that inspired our *Kineticolor* approach (represented by the “K”).

The field matured through the late 2010s, including Penn’s education-focused demonstration that smartphone cameras could capture concentration–time profiles in classical kinetics experiments such as the fading of crystal violet, enabling accurate determination of rate laws without spectrophotometers.^23^ Similar strategies were applied to quantify drying and browning processes in food systems, where RGB values were converted into standardized color spaces to derive kinetic models of chemical change.^24,25^ In an elegant study of the relative ease with which different reactors holding air-sensitive chemistries could be compromised by oxygen and moisture ingress, Bowring’s team used video recordings to quantify ketyl radical degradation.^26^ The results helped assess the integrity of various glassware stoppers and sealants. Cronin’s 2018 advancement in visual measurement of networked reaction systems,^27^ Hein’s demonstrations of process screening and monitoring using computer vision,^28,29^ Aspuru-Guzik’s approach to enabling team-based analysis in chemistry education,^30^ McKendry’s development of methods to assess protein binding and phase separations,^31^ and Ozcan’s approach to rheological characterization of films through image analysis of acoustic waves^32^ have pushed the envelope on what insights can be drawn on chemical processes from appropriate analysis of video footage. Parallel developments by Fitschen et al. in 2021 ^33^ and the teams of Kapur and Blacker in 2023 ^34^ revealed how image analysis could provide spatial and temporal insights into mixing processes that were previously inaccessible through conventional methods. These collective advances have, for us, inspired a principle that we have embodied in our *Kineticolor* approach:

If you can see it, the chances are that your camera (and computer vision methods) can help you measure it.

### 1.3 Trends and Broader Context

Our work on computer vision for chemistry maps onto a trend of increasing publication activity in the space (Figure 2). More broadly, chemical research and manufacturing sectors are experiencing a rapid digital transformation driven by three interconnected technological advances: workflow digitalization, laboratory automation, and AI-enhanced process control. From a market perspective, global chemical automation instruments sales are increasing with a 6% compound annual growth rate through 2028.^35^ Cost reduction, safety improvements, sustainable energy consumption, and regulatory compliance create compelling adoption drivers for this ongoing digital evolution across chemical manufacturing. In relation, a huge proportion of all AI-related chemistry publications have emerged in the past decade, reflecting the field’s rapid digital evolution.^36^ Industry 4.0 technologies have reached cost and performance levels enabling widespread chemical process integration.^37,38^ A critical challenge driving this digitalization is that 55% of stored chemistry data remains trapped in outdated systems, highlighting the need for integrated platforms that can automatically extract visual information.^39^

**Figure 2:**
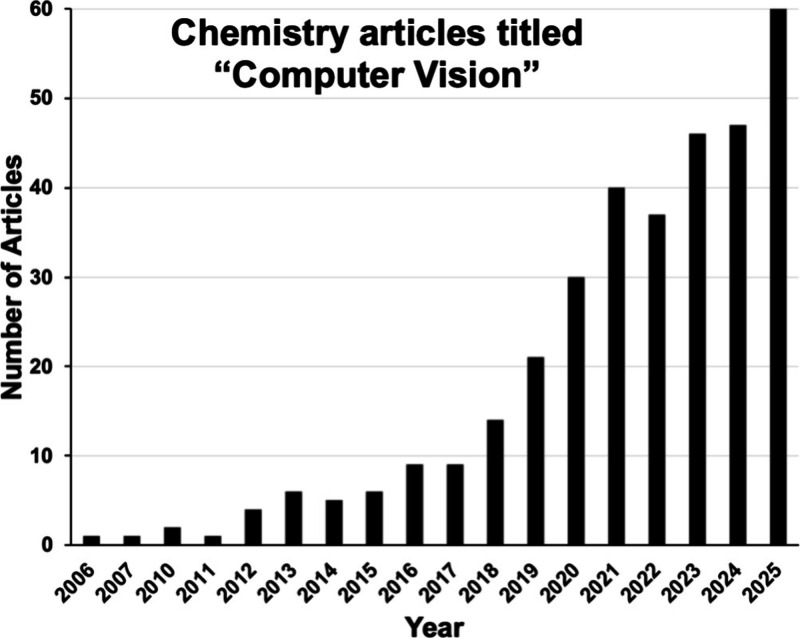
Number of primary research articles with “Computer Vision” in the title (2006–2025; English; Chemistry subject only; Scopus).

Beyond digitalization of large-scale manufacturing, self-driving laboratories and autonomous robotic platforms have fundamentally transformed chemical experimentation.^40–43^ Mobile robotic systems now autonomously execute exploratory synthetic chemistry while combining liquid chromatography-mass spectrometry (LC-MS) and benchtop nuclear magnetic resonance (NMR) capabilities.^44^ Such laboratories represent the natural evolution of the value derived from analytical technologies, featuring complete automation with adaptive optimization based on real-time feedback.^45^ Programmable chemical synthesis systems driven by chemical programming languages enable universal abstraction of synthesis operations, creating standardized environments where process monitoring tools can consistently monitor programmed chemical syntheses.^46,47^ Like a critical cog in a larger machine, computer vision serves as a promising technology toward digital transformation in these self-driving laboratories, providing noninvasive monitoring capabilities that integrate with broader robotics and AI frameworks.

It is worth noting that camera-based methods are not the first approach to extracting quantitative color data from chemical systems. Single-point colorimeters have a long history in chemical analysis, including the determination of rate constants and equilibrium parameters from absorbance measurements.^48^ Commercial colorimeters occupy an intermediate position in the cost hierarchy: more expensive than webcams but considerably cheaper than multiwavelength spectrometers. Camera-based approaches offer complementary advantages, including spatial resolution across the full reaction vessel, multiregion monitoring from a single device, and the ability to capture mixing and heterogeneity information that single-point instruments cannot provide. The methods described in this Account should therefore be understood as extending, rather than replacing, the established toolkit of colorimetric analysis. Indeed, other groups have applied computer vision and colorimetric approaches to diverse problems including inline flow titrations, gas dissolution monitoring, and continuous-flow extraction systems.^49–52^

### 1.4 Outline

Underpinning the digital chemistry mega-trend is a need for ever more sources of high-quality experimental sensor data. This Account chronicles our development of computer vision methods as one such source of chemical data. Readers will discover practical approaches for using cameras to convert everyday visual observations into kinetic data, along with implementation insights across diverse applications. The discussion provides actionable guidance for reaction monitoring while demonstrating how computer vision complements traditional analytical methods to extract previously inaccessible time-resolved information about chemical transformations.

## 2 Let There Be Light, Then Color Models

The story of bringing computer vision to chemical reaction monitoring begins with understanding a fundamental truth: what you see is not what your camera captures. This disconnect between subjective human perception and objective digital representation becomes the cornerstone of quantitative color analysis. When we embarked on developing *Kineticolor*, our first challenge was not building software, but rather learning to speak the mathematical language that cameras use to encode the light captured on their semiconducting rectangular sensors.

Consider the viral dress debate of 2015, where a single photograph sparked global arguments about whether a dress was white and gold or blue and black (Figure 3). This phenomenon perfectly illustrates how subjective human color perception can be, influenced by lighting conditions, individual physiology, and even psychological factors.^53^ For chemists seeking to monitor reactions through color changes, this subjectivity presents a significant challenge to interpreting lab book notes of old, where those serendipitous scribblings of qualitative by-eye observations live and die. How can we put numbers against the transformations we observe when our eyes might deceive us?

**Figure 3:**
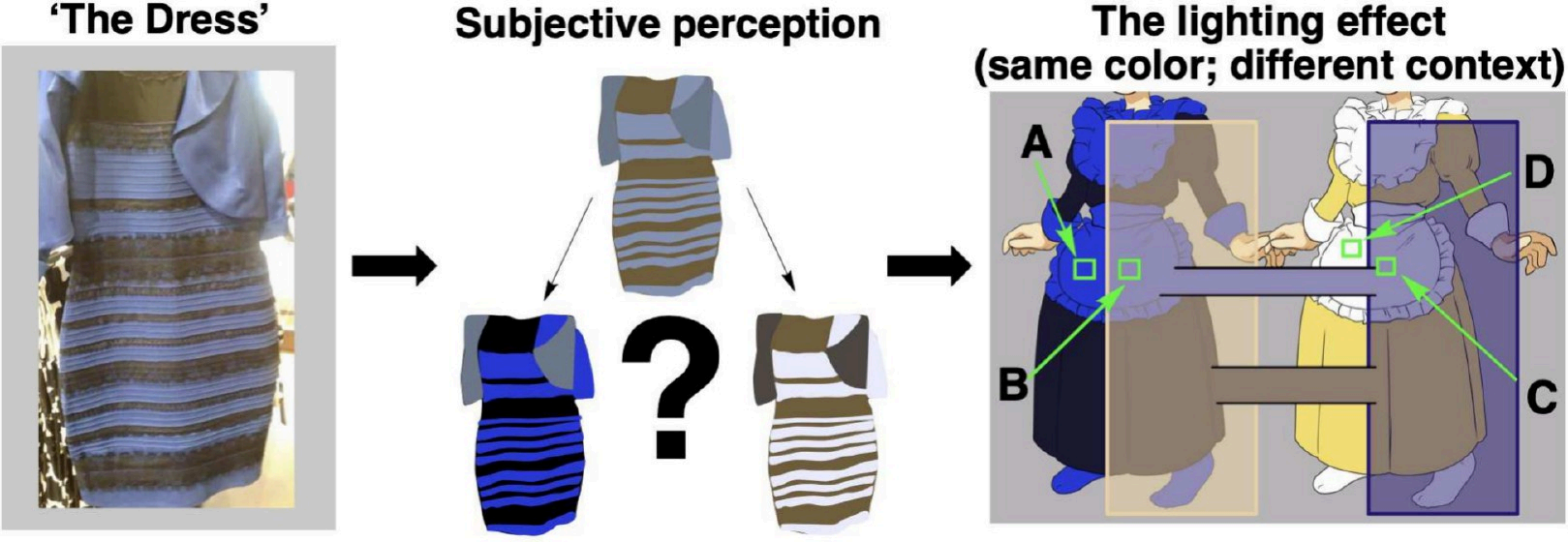
**Left**: The original image that sparked the dress debate. **Middle**: The heart of the debate - is the dress white and gold or blue and black? **Right**: A cartoonised depiction showing that, despite appearing different due to contextual lighting effects, patches B and C are mathematically identical in color, demonstrating the subjective nature of human color perception that necessitates quantitative computer vision approaches for reliable chemical analysis. See also: Table 1.

### 2.1 Color Spaces

To overcome subjective color perception exemplified by “the dress”, we need to transform color observations into objective, numerical representations. Digital cameras accomplish this by encoding the detected wavelengths of light in mathematical color spaces that separate lighting influences from chromaticity (Figure 4 and Table 1). At its most fundamental level, a digital image is a three-dimensional array of numbers, where each pixel contains values for red, green, and blue intensities. This RGB color space forms the foundation of digital imaging, but it is poorly suited to chemical analysis: the relationship between RGB values and perceived color is nonlinear and device-dependent, making it difficult to relate small numerical changes to meaningful chemical transformations.^15^ This limitation led us to explore alternative color representations. The CIE–XYZ color space serves as a device-independent intermediate from which we access various RGB color spaces that have proven invaluable for reaction monitoring. Then, from RGB, translation to the HSV (Hue, Saturation, Value) space separates color identity from brightness, which is useful when lighting conditions vary. The most powerful color space for quantitative chemical analysis, in our experience, is CIE–L*a*b* (also derived from CIE–XYZ). Designed to approximate human vision, it consists of three axes: L* (lightness), a* (green-to-red), and b* (blue-to-yellow). Its crucial advantage is perceptual uniformity: equal numerical distances correspond to approximately equal perceived color differences (Figure 4).

**Figure 4:**
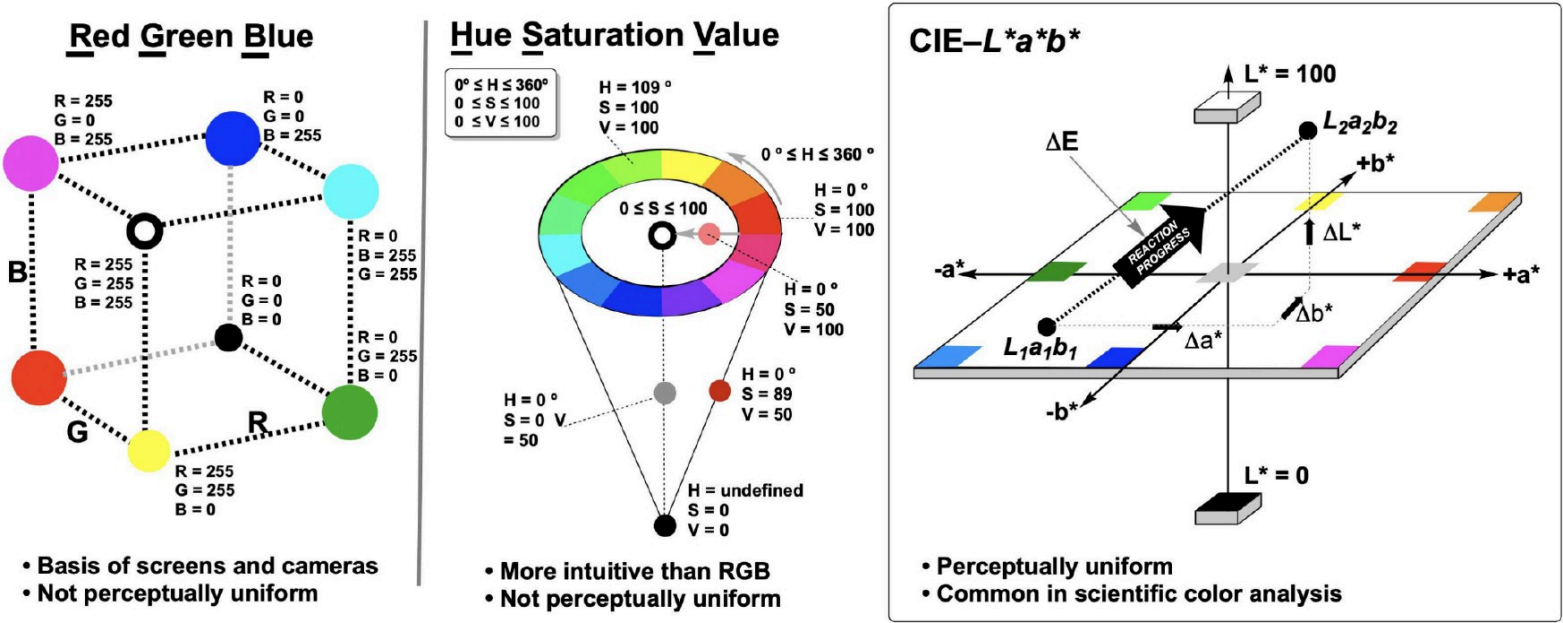
Simplified depiction of the RGB, HSV, and CIE–L*a*b* color spaces. Time-resolved data for all components of all color spaces are accessible via *Kineticolor* computer vision analysis.

**Table 1:** Average RGB, HSV and CIE–L*a*b* Pixel Values for Four 120 × 120 Square Regions of Interest on the Cartoon Apron under Each Light Source Shown in Figure 3

Region of Interest (ROI)	Average RGB	Average HSV	Average L*a*b*
A	[38, 59, 222]	[53, 211, 222]	[54, 43, −63]
B	[147, 140, 187]	[68, 64, 187]	[79, 17, −10]
C	[147, 140, 187]	[68, 64, 187]	[79, 17, −10]
D	[255, 254, 255]	[300, 1, 255]	[99, 8, 5]

### 2.2 Δ*E* and Derived Color Metrics

While individual color space coordinates provide valuable information, chemical reactions often involve complex color transformations that do not align neatly with single color axes or channel. Oftentimes, derived color metrics, based on calculations involving the individual color channels as inputs, can extend the analytical power of computer vision beyond simple visual observation, and enable the complexities of multiple color signals to be condensed into a single useful metric.

Inspired by researchers’ efforts to understand the complexities of food decomposition,^25,54^ this challenge led us to embrace Δ*E* (Delta E), a color-agnostic metric that captures the magnitude of color *change* regardless of the specific colors involved. Δ*E* originates from the CIE–L*a*b* color space and, in its simplest form, represents the Euclidean distance between two colors in this three-dimensional space (eq 1 and Table 2).^55^

1 $$\Delta E_{1976}=\sqrt{{\left(L_{2}-L_{1}\right)}^{2}+{\left(a_{2}-a_{1}\right)}^{2}+{\left(b_{2}-b_{1}\right)}^{2}}$$

**Table 2:** A Guide to Interpreting Δ*E* Values in Reaction Monitoring

Δ*E* Range	Interpretation/Observational Significance
<1.0	Imperceptible; within instrumental noise floor.
1–2	Threshold of perception; detectable by experienced observers.
2–10	Minor color shift; indicative of early stage reaction or low concentration.
10–25	Moderate change; readily identifiable as a progression in state.
25–50	Distinct color transition; high confidence in reaction advancement.
50–100	Major qualitative shift; typical of complete colorimetric transformation.
>100	Extreme deviation; comparison of disparate or polar opposite hues.

In our implementation, we calculate Δ*E* by comparing each video frame to the first frame, creating a time series that captures the cumulative color change throughout a reaction. A Δ*E* value of 0 indicates no color change, while values above 10 typically represent visually obvious transformations (Table 2). The beauty of this metric lies in its universality: whether monitoring the deep purple formation during a manganese oxidation or the subtle yellowing of an amide coupling reaction, Δ*E* provides a consistent scale for comparison, and is accessible to those who are color blind.^56^ When we monitored palladium catalyst degradation, we tracked not just the final black color of “Pd-black” formation, but the kinetics of this transformation from the yellow Pd­(II) precursor.^5^ The rate of change of Δ*E* correlated with catalyst deactivation rates, providing time-resolved feedback on reaction conditions that might compromise catalytic performance.

In balance, Δ*E* does possess some limitations in reaction monitoring. Primarily, the metric is highly sensitive to the choice of the initial reference frame. Furthermore, Δ*E* reduces a three-dimensional color transition into a single scalar value, which can mask intricate trajectories of color evolution. In reactions involving multiple intermediate species, the path through CIE–L*a*b* space is rarely linear. A scalar Δ*E* cannot distinguish between a direct chromatic shift and a complex, multistage evolution where the vector of change rotates or reverses, potentially leading to an underestimation of the true kinetic complexity occurring within the reactor.

Beyond Δ*E*, we have found value in alternative derived metrics. While investigating trityl dye decay processes as part of an investigation to enable color correction between smartphone cameras,^55^ the *RGB Sum Response* metric provided a simple measure of overall brightness change (eq 2, Figure 6). In this example, the condensed metric helped reveal a limitation in the palette of colors (i.e., gamut) that could be captured by the camera in use.

2 RGBSumResponse=765−(R+G+B)

**Figure 5:**
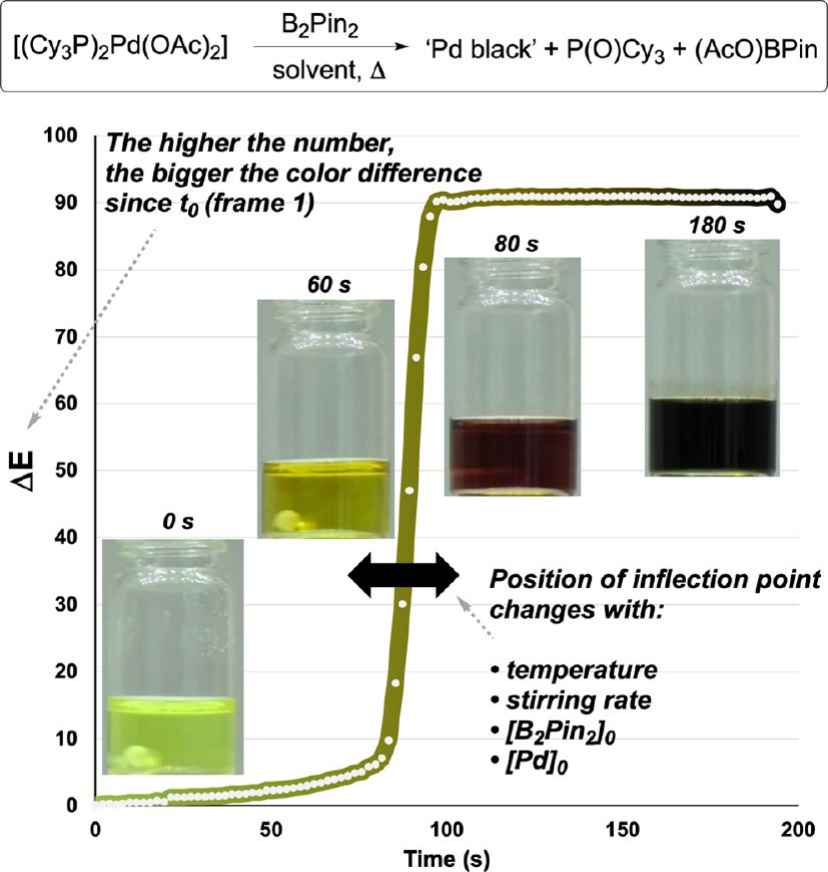
Computer vision monitoring of palladium catalyst degradation using Δ*E* color analysis. Visual transformation from yellow [Pd­(PCy_3_)_2_(OAc)_2_] precatalyst to black “Pd black” upon B_2_Pin_2_-mediated reduction under air, tracked using CIE–L*a*b* coordinates. Time-resolved Δ*E* profiles show concentration-dependent degradation kinetics, where Δ*E* represents the Euclidean distance in CIE–L*a*b* color space relative to the initial frame, providing a universal metric for quantifying color change independent of specific hues. White circles show captured data points; the colored gradient line is a guide to the eye alongside the still images.

**Figure 6:**
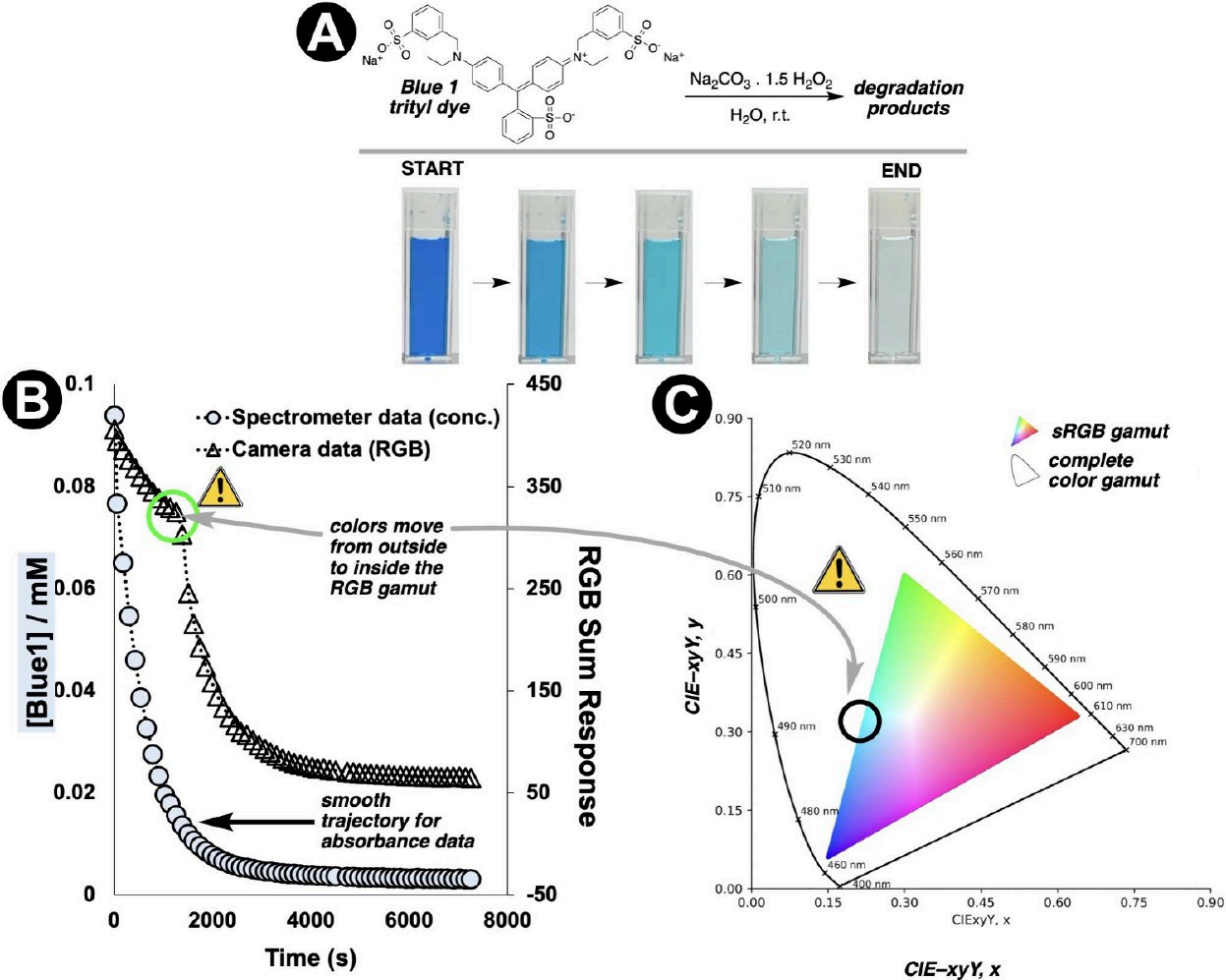
Comparison of spectrometer and computer vision monitoring of trityl dye degradation. **A**: Visual progression from blue to colorless during Na_2_CO_3_/H_2_O_2_-mediated degradation. **B**: Correlation between spectrometer-derived concentration data and RGB sum response (eq 2), demonstrating how brightness changes can quantify chemical conversion when color fades to colorless. **C**: The ‘shouldering’ effect in the RGB sum profile in **B** reveals color gamut limitations at high concentrations, where the quantified color is artificially clipped based on the range of sRGB colors (inner triangle) the camera’s sensor is able to quantify.

### 2.3 A Deeper Dive on the Added Benefit of Video versus Image Data

The transition from single-image colorimetric analysis^10^ to time-resolved video monitoring represents the most important conceptual leap in our approach. A video is simply a sequence of still images captured at regular intervals (typically 24–30 frames per second) and reaction kinetics are embedded in how these frames change over time. The ease with which video frames can be pruned or processed means the chemist has access to a tool that can capture both very slow and very fast transformations with a single recording (Figure 7).

**Figure 7:**
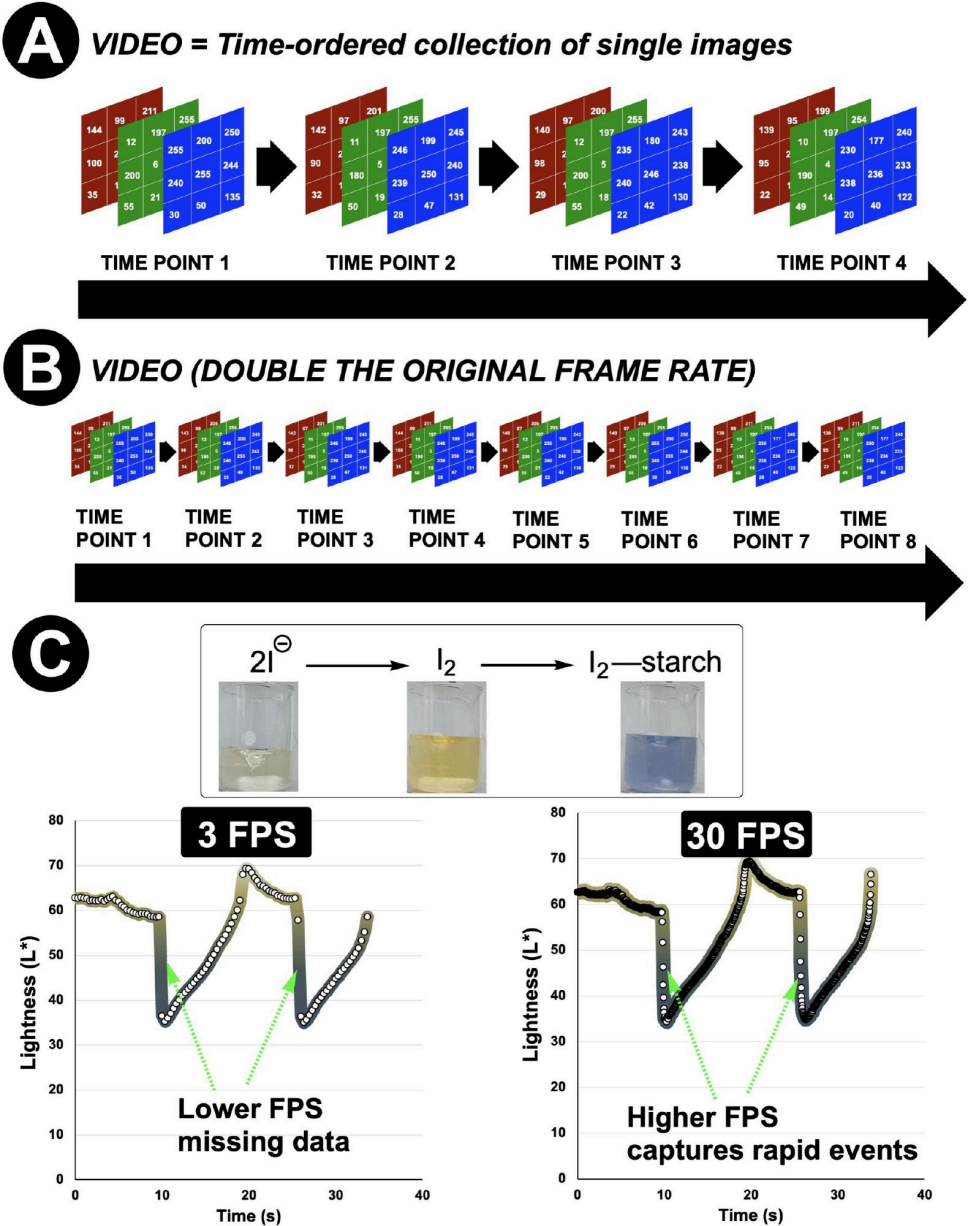
RGB digital image representation and temporal resolution. **A**: A video as a temporal sequence of image frames. **B**: The effect of increasing frame rate on temporal data density, critical for resolving fast reaction kinetics. **C**: An example of the consequences of changing video frames per second (FPS), using data from monitoring the Briggs–Rauscher iodine clock reaction, where a 10-fold increase in frame rate enables capture of the rapid iodine-starch complex formation event. Recording at higher frame rates preserves fast kinetic events that would otherwise be lost. In **C**, white circles show captured data points; the colored gradient line is a guide to the eye alongside the still images.

In *Kineticolor*, we average color values across user-selected regions of interest (ROIs) within each frame, reducing the influence of local variations from stirring or concentration gradients and focusing on bulk reaction properties. Frame rate selection can be adjusted retrospectively: reactions recorded at high frame rates can be analyzed at whatever temporal resolution proves most informative, from subsecond acid–base neutralizations to catalyst degradation processes spanning hours.

The kinetic information that can be extracted from video extends far beyond single-image approaches. We have demonstrated the ability to identify reaction onset times, measure rate constants, detect plateau regions indicating completion, and resolve multiple phases within complex reaction sequences. Video analysis also captures transient phenomena that periodic manual observation would miss. We have observed rapid color changes within seconds followed by slower evolution over hours, events that continuous video capture preserves. The spatial dimension adds further analytical power: by segmenting videos into multiple ROIs, we can monitor concentration gradients, mixing efficiency, and parallel reactions within a single experimental setup, as explored in subsequent sections.

It should be noted that Δ*E*, as a scalar metric, reduces the three-dimensional color trajectory to a single distance value, discarding information about the direction of color change. Additionally, Δ*E* values are reference-frame dependent: the choice of initial reference frame influences all subsequent measurements, which may complicate comparisons across experiments with different starting conditions.

## 3 Using Color to Monitor Bulk Transformations

With these principles established, we now illustrate how time-resolved color monitoring translates into practical chemical analysis across three diverse domains: mechanistic analysis in electrochemical reactions, monitoring catalyst lifetime, and forensic spot test development.

### 3.1 Alternating Electrochemical Potential

Spectroelectrochemistry has a rich history,^57^ and several groups have exploited color change as a readout for electrochemical processes. Prominent examples include thin-layer UV–vis cells for mechanistic elucidation^58^ and the use of electrochromic indicators for ion sensing.^59^ In a complementary fashion, smartphone colorimetry has been leveraged for point-of-need electroanalysis.^60^ Our entry point into this space was deliberately simple: tracking the visible color oscillations of a redox-active species in a small electrochemical cell.^61^

Our earliest exploration emerged from electrochemical systems, where dramatic color changes can accompany electron transfer processes. As a means of investigating the mechanistic implications of periodically flipping electrode potential in synthetic organic transformations, we monitored tris­(4-bromophenyl)­amine under alternating potential conditions, expecting a controlled colorless-to-blue transformation upon oxidation to its radical cation (Figure 8). Full-frame video was recorded throughout the experiment; rectangular regions of interest (ROIs) encompassing the solution were then defined within the *Kineticolor* software, and the spatially averaged pixel color within each ROI was extracted frame-by-frame for subsequent analysis.

**Figure 8:**
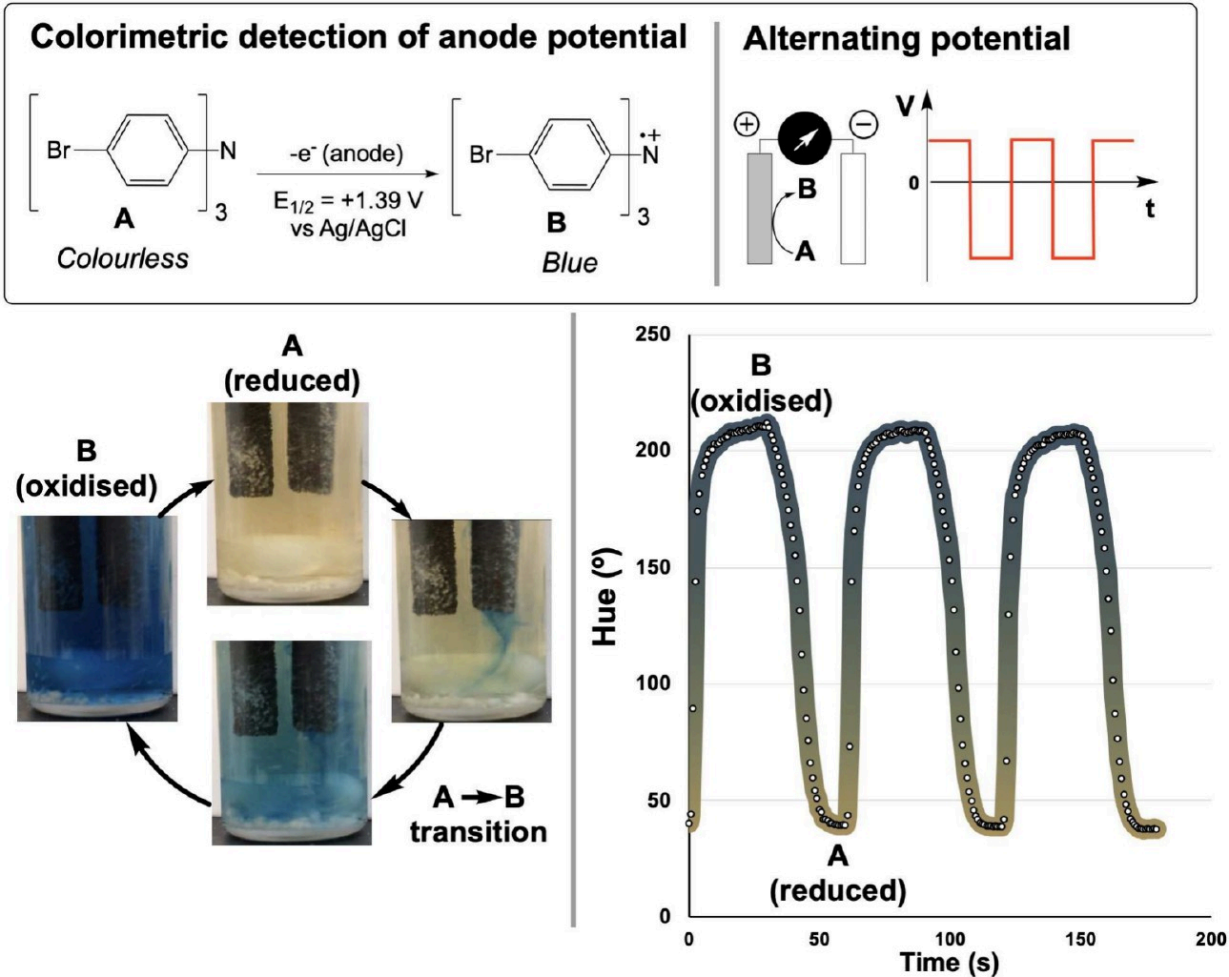
Noninvasive monitoring of radical cation formation during alternating potential electrochemistry. **Top**: Electrochemical oxidation of tris­(4-bromophenyl)­amine showing colorless to blue transition at *E*_1/2_ = 1.39 V vs Ag/AgCl. **Bottom**: HSV hue analysis reveals periodic oscillations between yellow (∼50°) and deep blue (∼212°) synchronous with 30-s potential alternation cycles, demonstrating how computer vision enabled detection of unexpected potential deviations in small-scale electrochemical cells where conventional probing would be impractical.

What followed illustrated the diagnostic power of computer vision in electrochemical systems. While programming the cell to alternate between oxidizing and reducing potentials every 30 s, we observed unexpected color changes revealing a systematic problem: as the electrochemical potential was alternating across the electrodes, it was not doing so symmetrically. Monitoring the hue channel of the HSV color space over time captured periodic oscillations between yellow (∼50°) and deep blue (∼212°) corresponding to the potential cycling. The time-resolved data revealed an appreciable deviation from the programmed working potential. The discovery had a profound impact on our understanding of the oxidation processes under study, which we would have missed if not for the use of a colorimetric tracer, sensitive to anodic oxidation, and video recording.

This early work demonstrated that computer vision could serve as an independent verification method when manual sampling of the reaction mixture proved impractical. It provided quantifiable visual feedback that complemented traditional current and voltage measurements.

### 3.2 Catalytic Reaction Monitoring

The use of color to monitor catalytic reactions has precedent across several domains. Visible color changes associated with transition-metal catalyst speciation have long served as qualitative indicators of reaction progress,^62,63^ and recent efforts have sought to quantify these observations. For example, smartphone-based colorimetric assays have been developed for monitoring catalyst activity,^64^ while fluorescent ligand design has been deployed to enable fluorescence monitoring in cross-coupling reactions.^65^ Our work in this area focused on the specific and visually infamous phenomenon: the formation of palladium black during aerobic Miyaura borylation.^5,62^

We next turned to one of the most persistent challenges in homogeneous catalysis: monitoring catalyst degradation. Palladium catalyst degradation to form “Pd black” is widespread in cross-coupling chemistry, yet traditional methods like NMR do not capture these visible bulk kinetics.^62^ The simplified case of watching isolated Pd­(II) precatalysts decay was introduced in Figure 5.

By monitoring Pd-catalyzed Miyaura borylation reactions under aerobic conditions, we captured the yellow-to-black transformation using Δ*E* analysis (Figure 9). Regardless of the specific conditions, “Pd black” formation produced a characteristic color change quantifiable by Δ*E* and other color metrics. We observed rapid initial color changes corresponding to catalyst activation, followed by slower degradation as Pd black formed, and observed quantitative correlations between Δ*E* values and product formation measured by offline nuclear magnetic resonance (NMR) spectroscopy and high-performance liquid chromatography (HPLC) over defined reaction windows, evidenced through mutual information analysis that captures both linear and nonlinear dependencies between color and conversion data.^5,8^ As with the electrochemical study, full-frame video was captured and user-defined ROIs encompassing the solution were spatially averaged per frame. The correlation between Δ*E* and conversion was strongest during the period of active color change, but diverged at later time points where Δ*E* approached a plateau while conversion continued to increase, consistent with color saturation preceding full chemical conversion. This allowed color changes to serve as a real-time proxy for catalyst performance over the kinetically informative window without interrupting the process. Perhaps most valuable was our discovery that breakdown of the Δ*E*–conversion correlation could indicate experimental problems such as air ingress in supposedly inert reactions. Such applications transform computer vision from a monitoring tool into a diagnostic system that flags deviations from expected behavior.^26^

**Figure 9:**
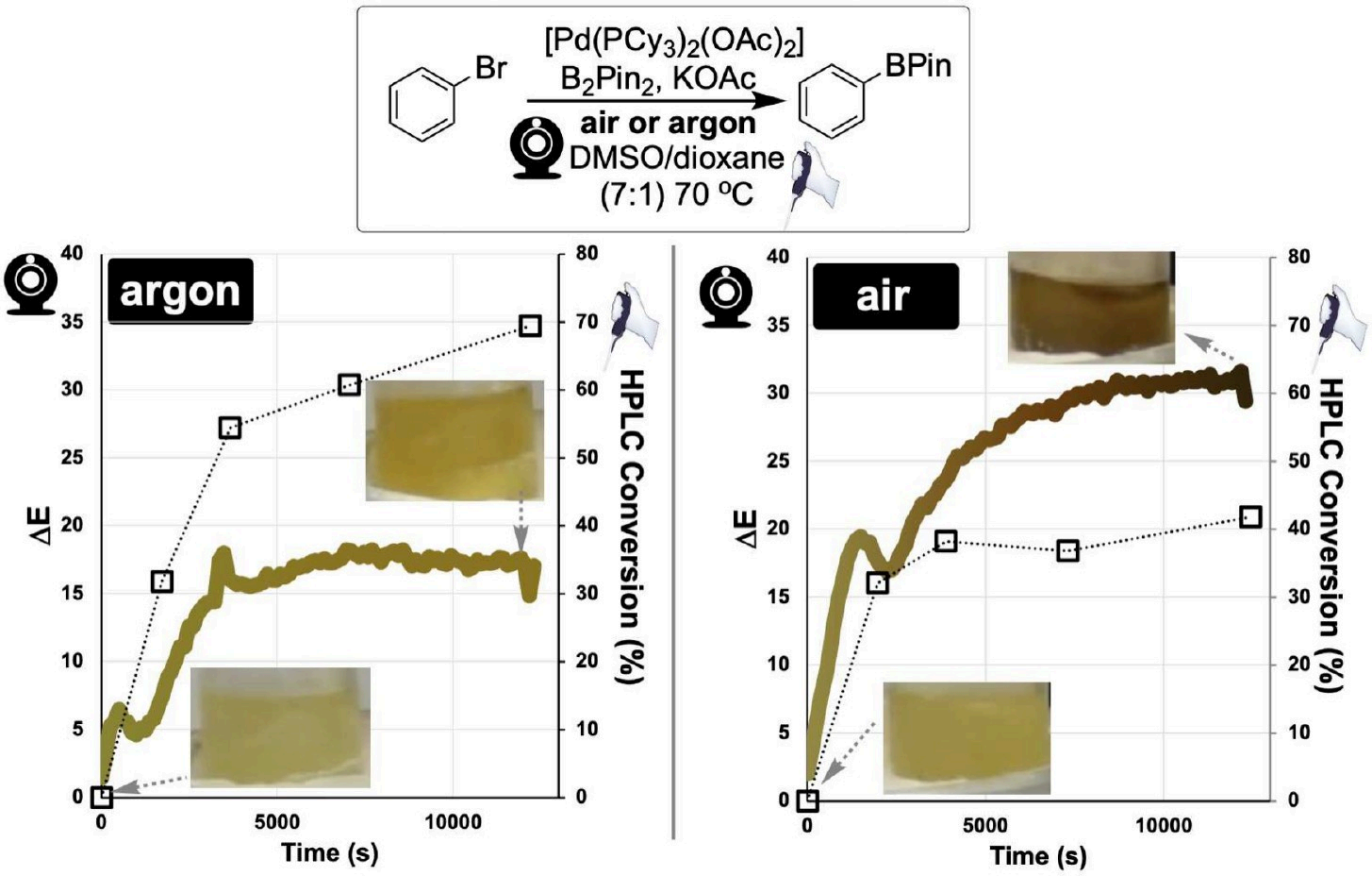
Computer vision monitoring of Pd-catalyzed Miyaura borylation. **Top**: Reaction scheme for aryl bromide borylation at 70 °C. **Bottom**: Time-resolved kinetic profiles comparing conversion to product (open squares) and Δ*E* color change analysis (gradient line). This approach enabled development of statistical analysis of the correlation between the color data and lower resolution sampling methods.^5^

### 3.3 Digitalizing Forensic Spot Tests

Colorimetric spot tests remain a mainstay of presumptive forensic analysis, valued for their simplicity, low cost, and rapid turnaround. Several previous efforts have explored digital enhancements to these classical methods, including smartphone-based color quantification for drug screening, machine-learning classification of colorimetric responses, and paper-based microfluidic devices with integrated color readout.^67–73^ Our contribution builds on this broader trend by introducing kinetic profiling as an additional discriminatory dimension.^7,74^

Traditional presumptive tests for illicit substances rely on subjective visual interpretation of color changes, leading to inconsistent results and limited specificity. Our initial work with amphetamine detection^7^ exemplifies how time-resolved color analysis can enhance both the objectivity and discriminating power of these established methods (Figure 10). However, several other controlled substances produce similar final colors, making visual discrimination challenging. Our computer vision approach addresses this by capturing not just the final colors, but the kinetics of color change. The Δ*E* versus time profiles revealed unique temporal signatures for each substance–reagent combination: while two drugs might produce visually similar red colors with the Mandelin reagent, for example, their color development kinetics often differ dramatically (Figure 10).

**Figure 10:**
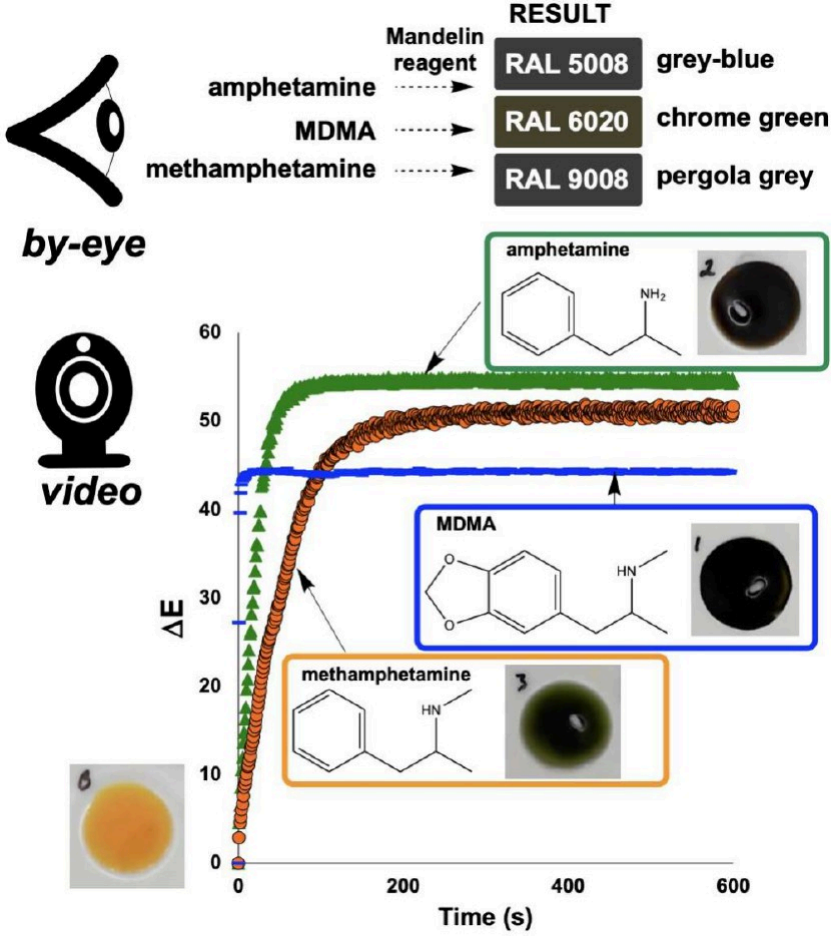
Time-resolved computer vision analysis reveals kinetic differences in forensic Mandelin spot tests despite similar visual end points. **Top**: By-eye observations show amphetamine, MDMA, and methamphetamine all produce similar gray-blue to brown color changes (RAL color codes shown). **Bottom**: Δ*E* kinetic profiles demonstrate distinct reaction rates despite visually similar end points: MDMA (fastest, blue line), amphetamine (intermediate, green triangles), and methamphetamine (slowest, orange circles), providing quantitative kinetic fingerprints for enhanced forensic discrimination.

More recently, when exploring tests for the veterinary drug xylazine (which presents an acute problem in drug abuse, at the time of writing),^75^ we exposed it to Marquis, Mandelin, and Mecke reagents; xylazine produced distinct color changes: yellow, blue, and red, respectively.^74^ Systematic evaluation across multiple controlled substances demonstrated that the combination of three time-resolved color tests could provide reliable xylazine identification, eliminating subjective interpretation while increasing discriminating power beyond what traditional spot testing could achieve.^75^

This work illustrates a broader principle: established colorimetric methods can often be enhanced through digital transformation rather than replacement, preserving simplicity while adding quantitative rigor.

## 4 Accelerated Discovery and Optimization

Beyond monitoring, computer vision can actively guide process improvement. We present two complementary approaches: plateau detection algorithms for optimizing individual reaction times, and parallel monitoring for accelerating high-throughput discovery.

### 4.1 Amide Coupling and Peptide Synthesis

Monitoring solid-phase peptide synthesis (SPPS) in real time has attracted attention from several directions.^76^ Reported approaches rely on UV–vis absorbance of cleaved chromophores (e.g., the Kaiser or TNBS tests) performed offline,^77^ while more recent innovations include on-resin FTIR monitoring,^78^ changes in refractive index,^79^ and UV–vis in flow.^80^ Camera-based color tracking offers a complementary approach that preserves the simplicity of visual indicators while adding temporal resolution.

SPPS provided an ideal proving ground for our approach. SPPS relies on repetitive amide coupling reactions with conservatively fixed reaction times (2–3 h per step). By monitoring the yellow color development from OAt anion release during hexafluorophosphate azabenzotriazole tetramethyl uronium (HATU)-mediated couplings, we captured videos whose kinetic information revealed when reactions actually achieved completion (Figure 11). The key innovation was implementing plateau detection algorithms that identified when the rate of color change fell below a defined threshold, indicating the reaction end point.^6^

**Figure 11:**
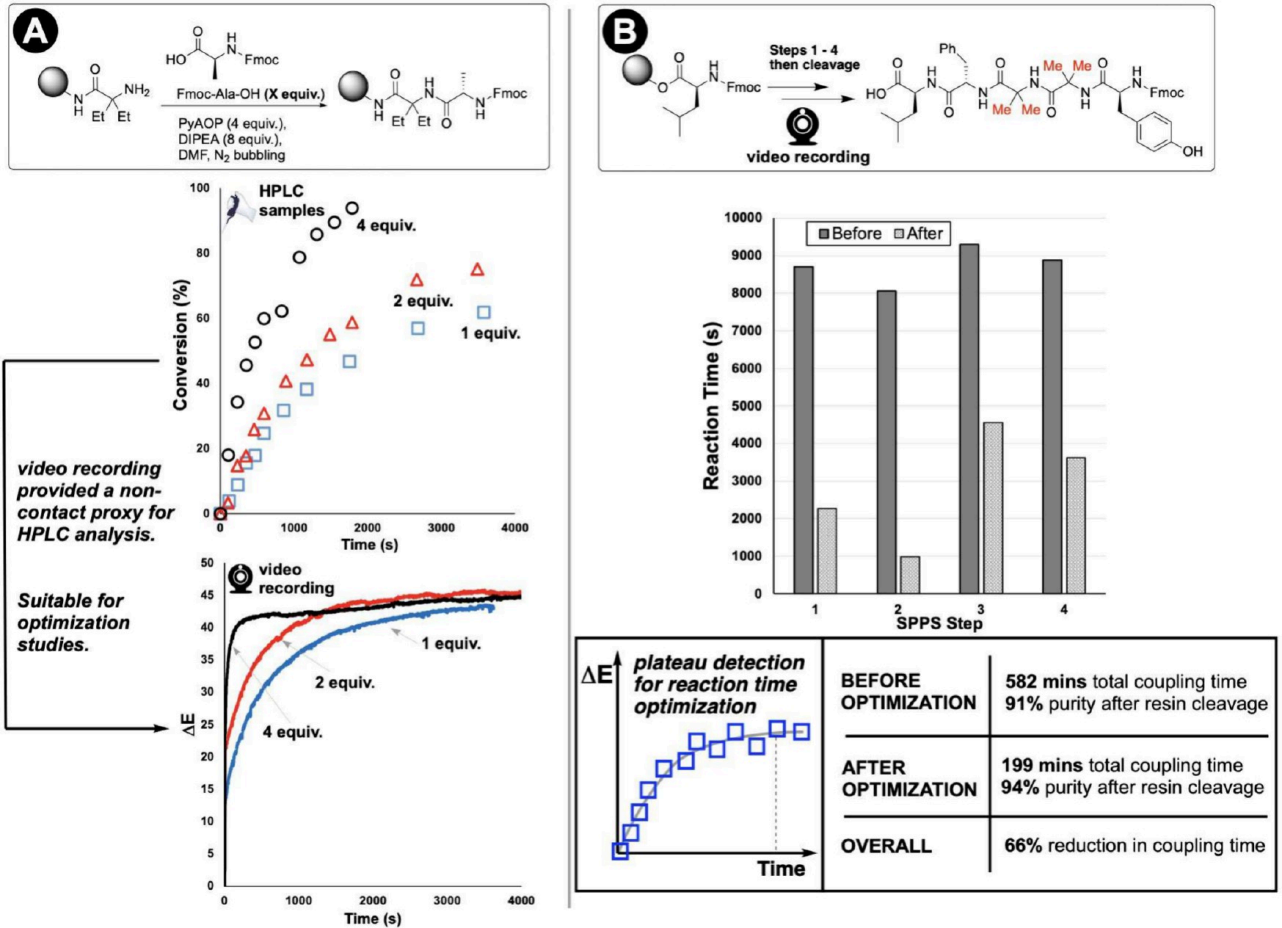
Computer vision enables concentration-dependent optimization of solid-phase peptide synthesis (SPPS). **A**: HPLC analysis of remaining substrate in solution shows concentration-dependent reaction kinetics, and corresponding Δ*E* profiles from video recording demonstrate how computer vision captures the same kinetic trends as HPLC noninvasively. **B**: Video recording enabled optimization of reaction times without interrupting the synthesis process or requiring resin cleavage for analysis.

The results proved significant. For a tetra- and, later, higher order peptides, plateau detection in Δ*E* data identified that most coupling steps reached completion within 30–45 min rather than the 2–3 h typically employed (Figure 11B). This insight enabled a 66% reduction in overall synthesis time while maintaining comparable yields and purity. Similar savings were achieved for a nine-residue peptide (KLLQDILDA, 51% reduction).^6^

Our approach also revealed structure–activity relationships between amino acid sterics and coupling kinetics. Correlating Δ*E* temporal profiles with computationally derived Sterimol B1 parameters demonstrated that bulkier amino acids exhibited predictably slower color development rates, providing a quantitative framework linking macroscopic color change to molecular-level steric effects. Validation in both manual and automated SPPS instruments confirmed the practical utility: computer vision-guided conditions produced comparable isolated yields while dramatically reducing processing time.

### 4.2 Parallel Reaction Monitoring

High-throughput experimentation (HTE) has transformed reaction discovery and optimization.^81,82^ Computer vision evidence a potentially complementary approach to the largely chromatography-driven analytics^83^ for reaction arrays where color change provides a convenient observable, enabling simultaneous monitoring of multiple wells without dedicated analytical instrumentation.

The evolution from single-reaction monitoring to parallel analysis represents our most significant scaling achievement.^84^ We developed multiple-ROI capabilities within *Kineticolor*, enabling simultaneous kinetic analysis of dozens of reactions from a single video recording (Figure 12).^8^

**Figure 12:**
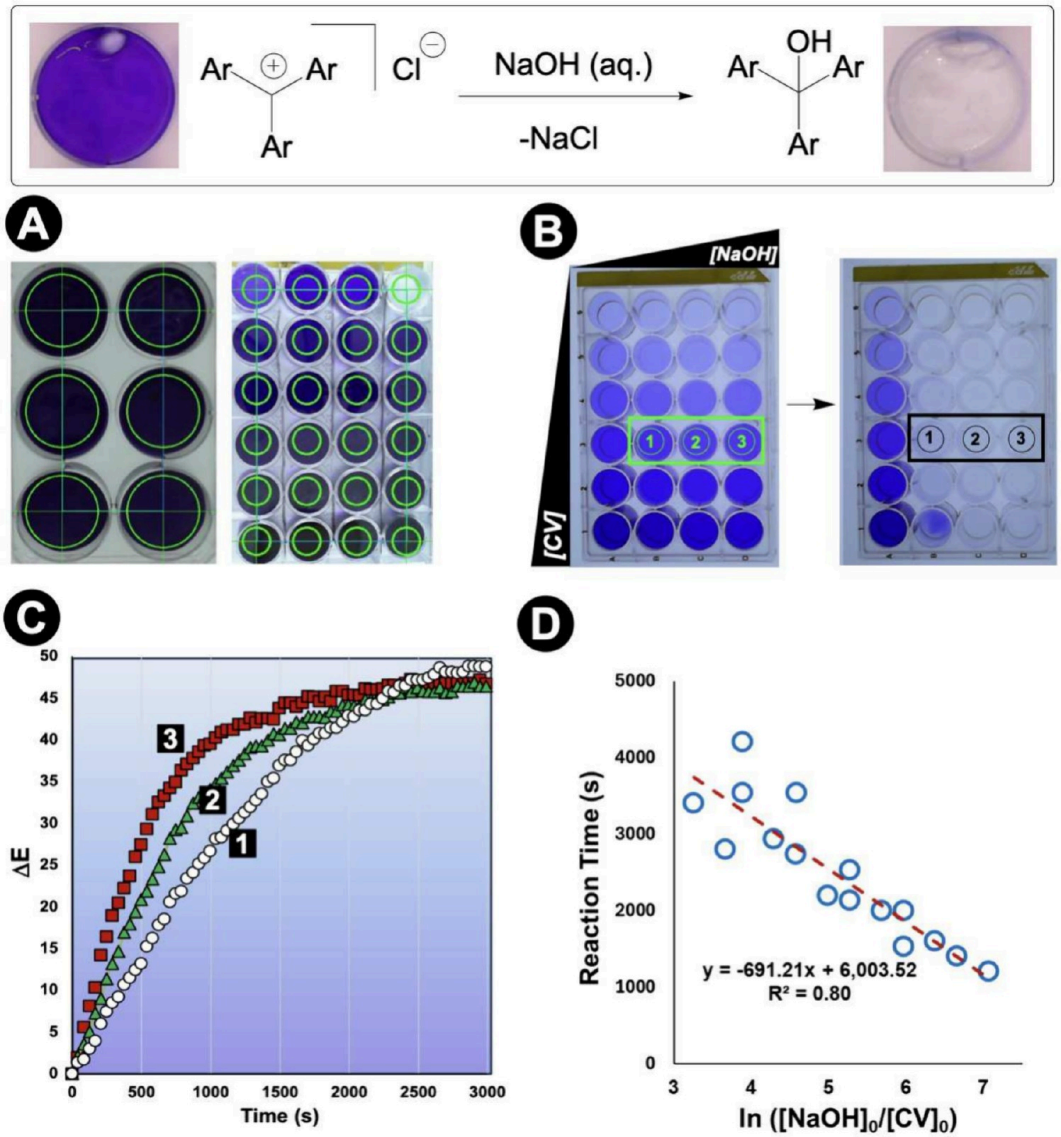
Parallel reaction monitoring with computer vision. **Top**: Crystal violet hydroxylation reactions demonstrate the capability to extract kinetics from multiple reactions simultaneously. **A**: Software setup, showing flexible ROI selection for different well plate dimensions. **B**: Reaction progression across a 24-well plate, for varying concentrations of crystal violet and hydroxide. **C**: Extracted kinetics from the independent wells highlighted in **B**. **D**: Summarized kinetic analysis from all 24 wells, based on plateau detection in Δ*E* vs time plots. The deviation from linearity at lower [NaOH]:[crystal violet] ratios represent the transition from pseudo-first to second order conditions.

Proof-of-concept studies using crystal violet hydroxylation across 24-well plates demonstrated this approach. Systematically varying reactant concentrations across wells generated comprehensive kinetic data sets revealing concentration-dependent kinetics and plateau times from a single video recording. The parallel capabilities also addressed mixing uniformity: charging identical conditions across all wells while varying stirrer bar sizes revealed significant kinetic variations reflecting nonuniform mixing.^8^ This demonstrates a diagnostic capability for identifying systematic experimental biases that might compromise high-throughput data quality.

Application to 4-dimethylaminopyridine (DMAP)-catalyzed esterification reactions demonstrated broader utility. Combining computer vision kinetics with offline HPLC analysis and mutual information statistics established quantitative correlations between color parameters and reaction conversion across multiple parallel reactions.^8^

The implications for computer vision on high-throughput discovery workflows are substantial, and others have expanded on the idea.^84–87^ Rather than requiring individual analytical measurements for each reaction in a screening campaign, parallel computer vision monitoring provides simultaneous kinetic profiling across entire plates while detecting systematic variations in mixing and temperature gradients that might otherwise compromise experimental validity. The potential scalability of this approach positions it as a natural complement to molecularly specific high-throughput techniques like LC-MS, offering temporal resolution and bulk reaction insights that guide optimization strategies and flag experimental anomalies.

A practical limitation of parallel monitoring is the trade-off between spatial resolution and well density. As the number of wells increases, the pixel area per well decreases, reducing the signal-to-noise ratio for color measurements in each individual reaction. This constraint places an upper bound on the plate density that can be monitored with a given camera resolution and field of view.

## 5 Mixing Analysis: Bridging the Language of Chemists and Chemical Engineers

Mixing characterization is a longstanding challenge in chemical engineering, and a variety of experimental and computational methods have been developed. Planar laser-induced fluorescence (PLIF),^88^ particle image velocimetry (PIV),^89^ and electrical resistance tomography (ERT)^90^ are among the established techniques for interrogating mixing fields. Computational fluid dynamics (CFD) simulations provide complementary spatial resolution.^91^ Our approach differs in its emphasis on accessible, camera-based methods that extract mixing metrics from standard video recordings of transparent vessels.

When we began discussing mixing with industrial collaborators, we were struck by a fundamental disconnect. Chemical engineers spoke fluently about Reynolds numbers, impeller geometries, and macro- versus micromixing. Meanwhile, synthetic chemists (we, by primary training, included) tended to treat mixing as an afterthought: “just stir faster” or “use a bigger stir bar”. This disciplinary divide, often bridged by process chemists turning discovery routes into scale-up routes, became the catalyst for what we now consider one of our most impactful contributions: democratizing mixing analysis through computer vision.^92,93^

The reality is that mixing matters on all scales, from milliliter vials to multitonne manufacturing vessels. Yet most tools for quantifying mixing remain in the domain of chemical engineering, and the reaction monitoring probes familiar to chemists can disrupt the very flow patterns they aim to measure. The act of measuring may itself change the kinetics. Computer vision offered us a rare opportunity to create a scale-independent, noninvasive approach that could speak to both disciplines.

Our journey began with a simple observation: if mixing affects reaction outcomes, and reactions often involve color changes, then analyzing those color changes over space and time should reveal mixing behavior. This insight led us to extend *Kineticolor* beyond color averaging to incorporate spatially resolved analysis, enabling cameras to ‘see’ mixing the way chemical engineers think about it, from overall mixing time down to mesomixing regimes and microscale eddies.

### 5.1 From Color Averaging to Area Segmentation

The most intuitive extension of our color averaging approach was to divide reaction vessels into multiple regions of interest and track each separately. Rather than treating a 5-L stirred tank reactor as a single averaged pixel, we began partitioning videos into grids, analyzing mixing row by row, column by column, and cell by cell (Figure 13). In well-mixed systems, color changes occurred synchronously across all regions. Poor mixing, however, created a characteristic ‘patchwork’ of color evolution, with some regions changing rapidly while others lagged behind. This approach helps chemists quantify how a reaction proceeds at different rates in different regions of the same reactor.

**Figure 13:**
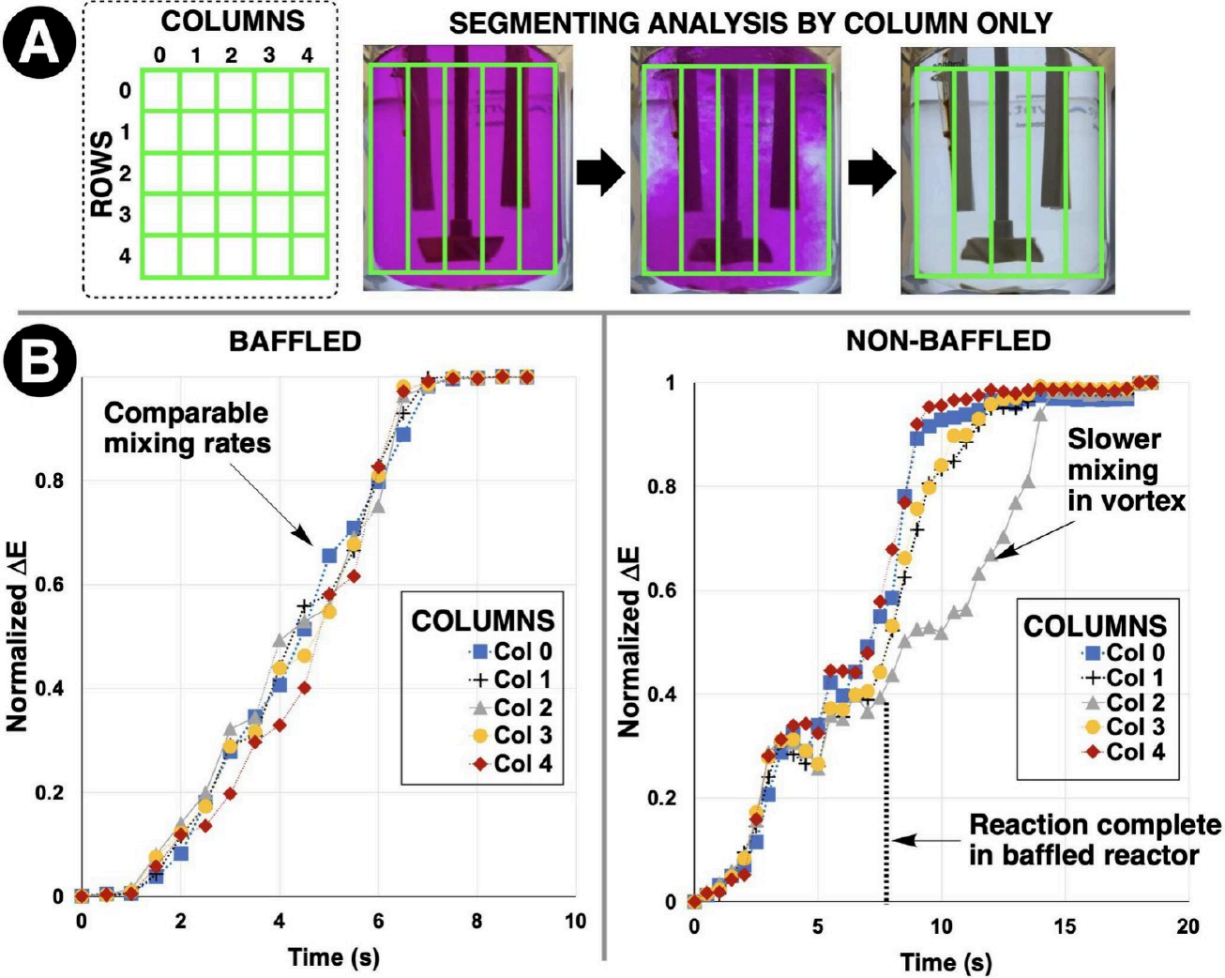
Cell- (or grid-)­based partitioning of a reactor vessel, with color evolution over time for different spatial regions during a mixing process. In this example, partitioning Δ*E* analysis by column helped quantify the vortex-induced mixing asymmetry in the nonbaffled reactor. Adapted with permission from ref 94. Copyright 2024 ACS Publications.

What was often deferred to chemical engineers and scale-up could now be quantified with the chemist in mind. We validated this approach using model systems, namely pH titrations where acid–base neutralization created predictable color fronts. By comparing our segmented analysis with probe-based mixing analysis and computational mixing models (more on this later), we demonstrated that spatial color variance provided a reliable proxy for mixing efficiency.^94^ More importantly, the same camera setup that worked for a 25 mL round-bottom flask could be scaled directly to multiliter pilot plants, providing continuity across the development pipeline.

The segmentation approach proved particularly valuable for understanding mixing heterogeneity in viscous systems.^95^ When working with deep eutectic solvents (DES), we observed that conventional mixing assumptions broke down. Regions that appeared well-mixed by traditional metrics showed persistent spatial variations when analyzed through our segmented approach.

### 5.2 Going Further with Segmentation through Contact Analysis

While spatial segmentation provided intuitive insights, it still relied on color averaging within each region. We realized that truly understanding mixing required analyzing the spatial relationships between pixels. This led us to implement *Contact* analysis,^96,97^ a technique originally developed for powder mixing that we adapted for liquid systems.^4^ The method involves converting each video frame to black and white using a user-defined threshold, then measuring the perimeter between black and white regions. Assuming a uniform background behind the reactor being video recorded, high *Contact* values indicate heterogeneous mixing; low values suggest either unmixed or fully mixed states (Figure 14).

**Figure 14:**
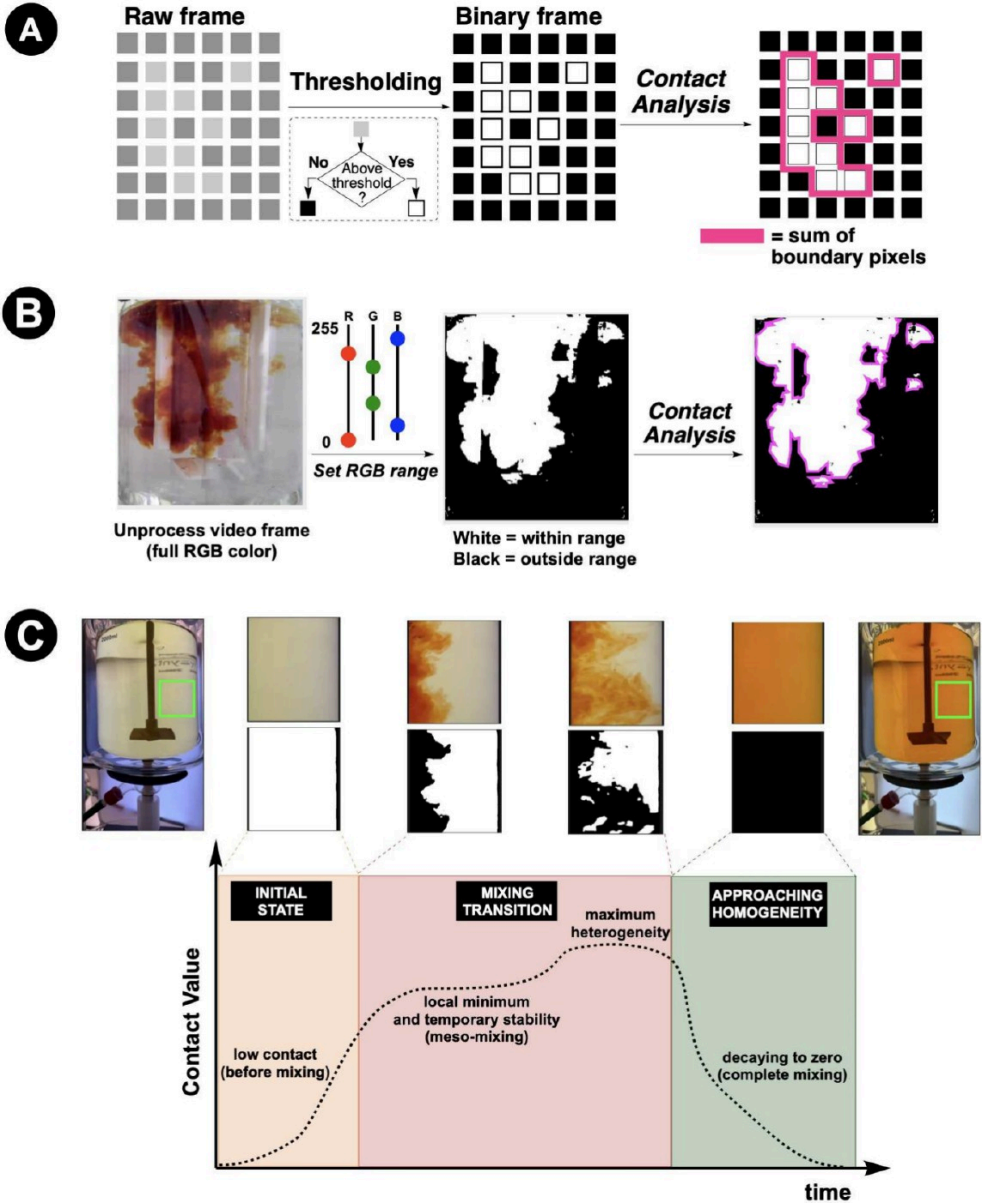
*Contact* metric for spatially resolved mixing analysis. **A**: Conceptual illustration: a grayscale image is binarized via pixel-intensity thresholding, and the total perimeter of white–black boundary regions is summed to yield the *Contact* value. **B**: Application to a reactor video frame: user-defined RGB thresholds convert a full-color image into a binary mask, from which *Contact* is extracted along the interfacial boundary (magenta). **C**: Time-resolved *Contact* analysis of a stirred mixing process. At the initial state, *Contact* is low (no interfacial area). A local minimum during meso-mixing precedes the peak at maximum heterogeneity, after which *Contact* decays toward zero as the system approaches homogeneity, revealing mixing dynamics that spatially averaged color metrics such as Δ*E* cannot resolve. Adapted with permission from refs 4 and 94 . Copyright 2022 and 2024 ACS Publications.

*Contact* analysis revealed mixing phenomena invisible to averaged color metrics, and increased spatial sensitivity beyond merely cell-based averaging of color. Small changes in impeller speed or geometry that produced barely detectable differences in average color created dramatic changes in *Contact* evolution. During the intermediate stages of mixing, *Contact* values peaked as swirling created maximum interfacial area between mixed and unmixed regions. This provided a quantitative signature for the mixing process itself, at the pixel level. From here, we pushed the sensitivity of time-resolved mixing analysis from video data one step further···

### 5.3 Texture Analysis for Time-Resolved Mixing Metrics

Our most sophisticated mixing analysis capability emerged from implementing time-resolved gray-level co-occurrence matrix (GLCM) analysis, a texture analysis technique from image processing that we adapted for real-time reaction monitoring. While the mathematical details might seem daunting, the underlying concept is intuitive: GLCM analysis quantifies the spatial relationships between neighboring pixels to characterize image texture.^97,98^

GLCM analysis is based on constructing a count-based matrix derived from summarizing the number of instances of defined pixel value pairs occurring in the input grayscale image (Figure 15A). Several complementary metrics are generated from the GLCM, the choice of which, like color spaces, can be a matter of preference depending on what provides the most intuitive means of process quantification for the application at hand. *Homogeneity* quantifies how similar neighboring pixels are to each other; high values denote greater similarity. *Angular second moment* (ASM) reflects pattern regularity, while *Entropy* measures randomness. Together, these metrics (and others derived from the GLCM approach) provide a multidimensional description of mixing state that captures subtleties invisible to simpler approaches (Figure 15B). The more subtle vector and scale dependence of GLCM metrics lies beyond the scope of this Account.

**Figure 15:**
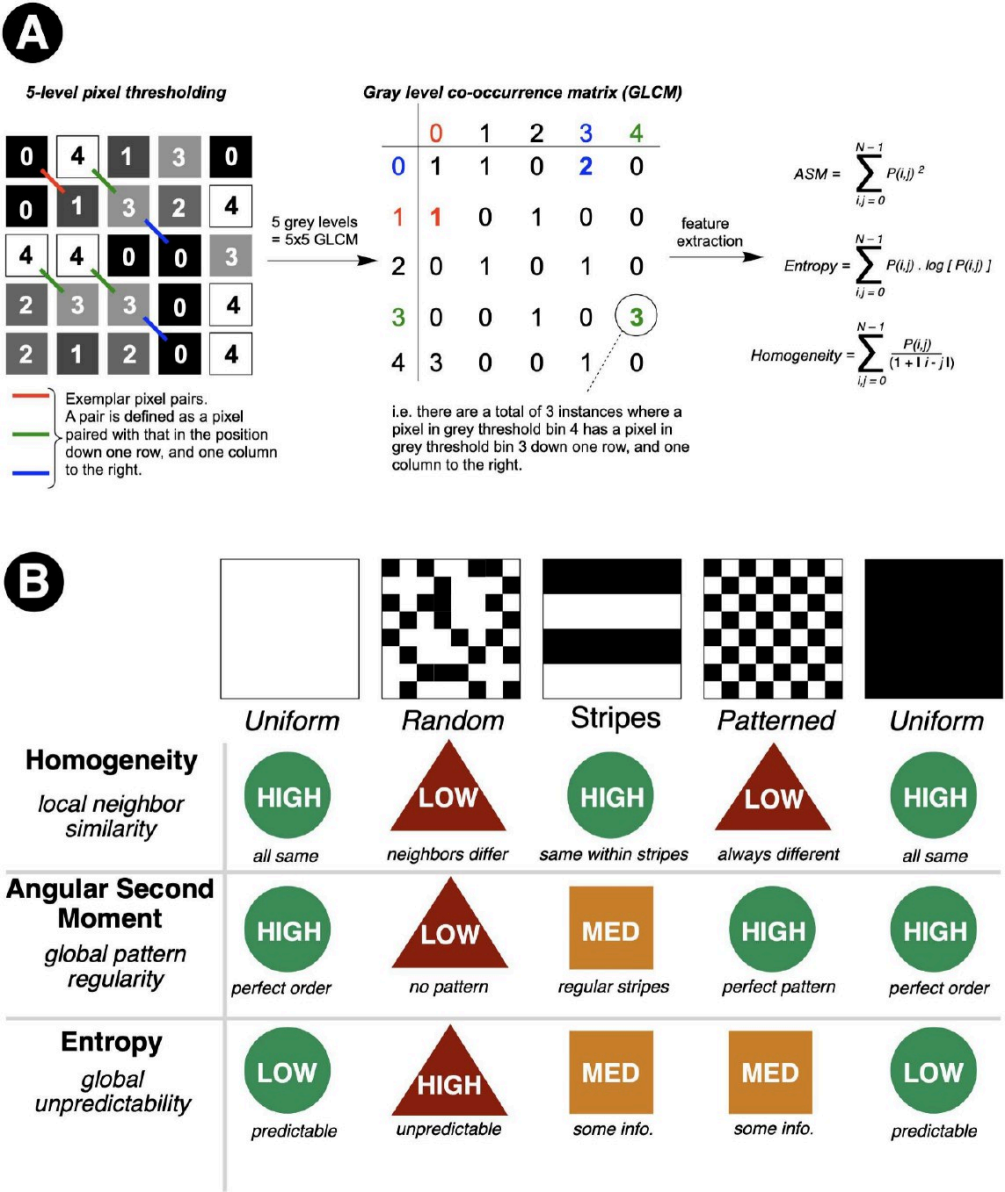
Gray-level co-occurrence matrix (GLCM) texture analysis. **A**: Construction of a GLCM from a 5-level grayscale image: pixel pairs (defined by a fixed spatial offset – e.g., one row down and one column right) are tallied into a vote-based co-occurrence matrix, from which texture features including Angular Second Moment (ASM), Entropy, and Homogeneity are extracted. **B**: Simplified interpretation of GLCM features across five archetypal texture patterns. Homogeneity measures local neighbor similarity, ASM quantifies global pattern regularity, and Entropy captures unpredictability. Notably, both uniform white and uniform black images yield identical GLCM feature values, confirming that these texture metrics are invariant to absolute gray level and instead characterize spatial structure, making them well suited for quantifying mixing states independently of the specific colors involved. Adapted with permission from refs 4 and 95 . Copyright 2022 and 2025 ACS Publications.

The power of the GLCM methodology became apparent when analyzing mixing times in viscous versus nonviscous solvent formulations, where traditional mixing metrics often failed. At elevated temperatures, these systems appeared well-mixed by eye and by averaged color analysis, yet GLCM metrics revealed persistent microscale heterogeneity: entropy values remained elevated while homogeneity values confirmed that neighboring pixels still differed significantly. Perhaps most importantly, GLCM analysis provided a shared quantitative language for describing mixing that bridged the chemist–engineer divide. Rather than debating whether a reaction looked well-mixed, we could point to specific texture metrics and their evolution over time (Figure 16).

**Figure 16:**
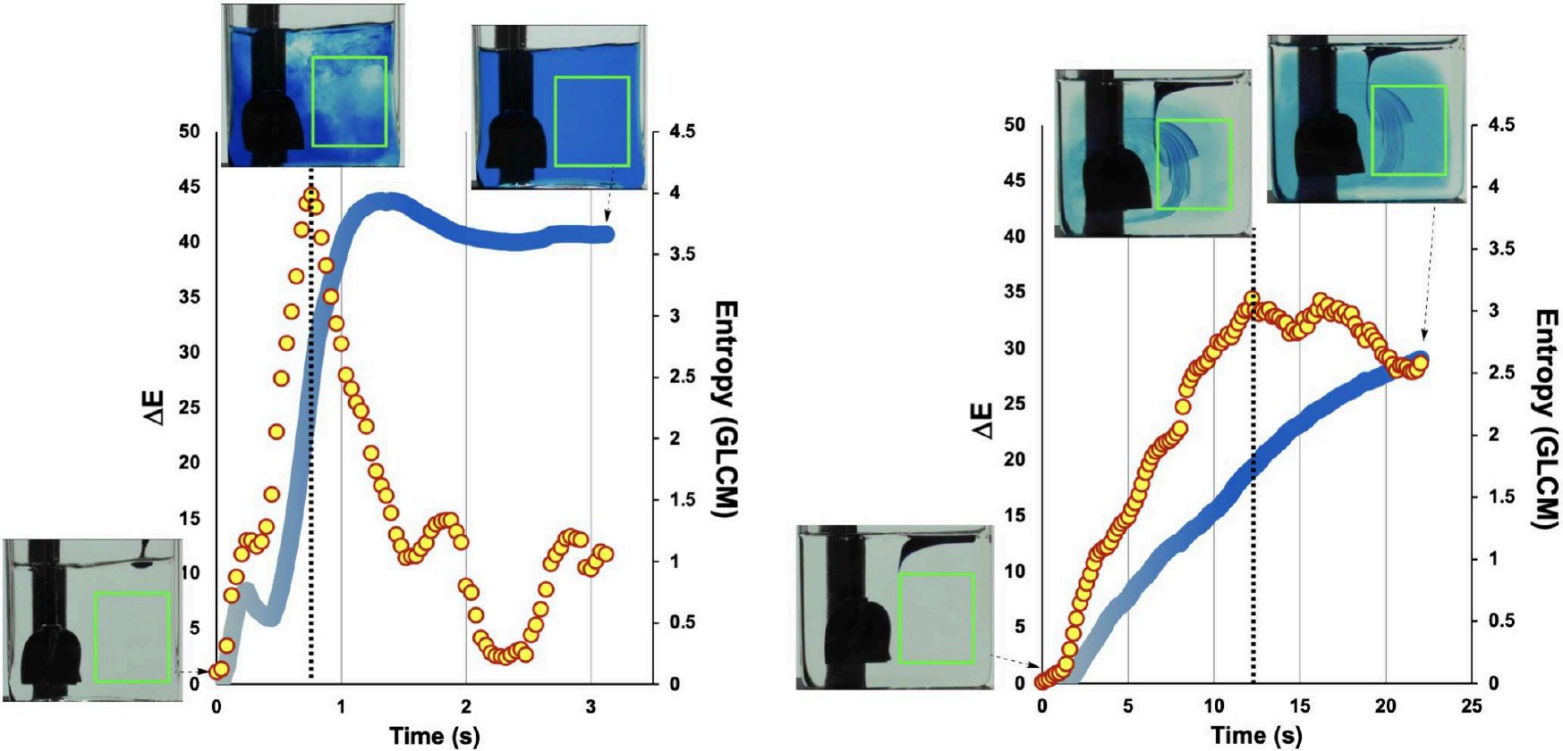
GLCM texture metrics capture spatially sensitive mixing analysis in the structure of the time series, complementing average pixel color metrics. This example shows that average Δ*E* shows maximum rate of change at the time point where entropy peaks, signaling the point of maximum heterogeneity.

### 5.4 Verifying Computational Fluid Dynamics Models

Armed with these spatially resolved metrics, we turned to a question that chemical engineers had been asking us since we first presented our mixing work: could computer vision provide experimental validation for computational fluid dynamics (CFD) simulations? CFD models are widely used to predict flow patterns and mixing behavior in stirred tank reactors, yet experimental verification remains a persistent challenge.^89,91,99–103^ Traditional validation relies on point measurements from invasive probes that, as discussed above, may themselves perturb the flow fields they aim to characterize.^94^

We approached this challenge using phenolphthalein-based acid–base titrations in transparent stirred tank reactors, capturing the spatial progression of the pink-to-colorless transition as a direct readout of mixing evolution. By comparing our spatially resolved Δ*E* profiles, Contact metrics, and GLCM-derived entropy values with CFD-predicted velocity fields, we could assess whether simulations faithfully reproduced the experimentally observed mixing dynamics (Figure 17).

**Figure 17:**
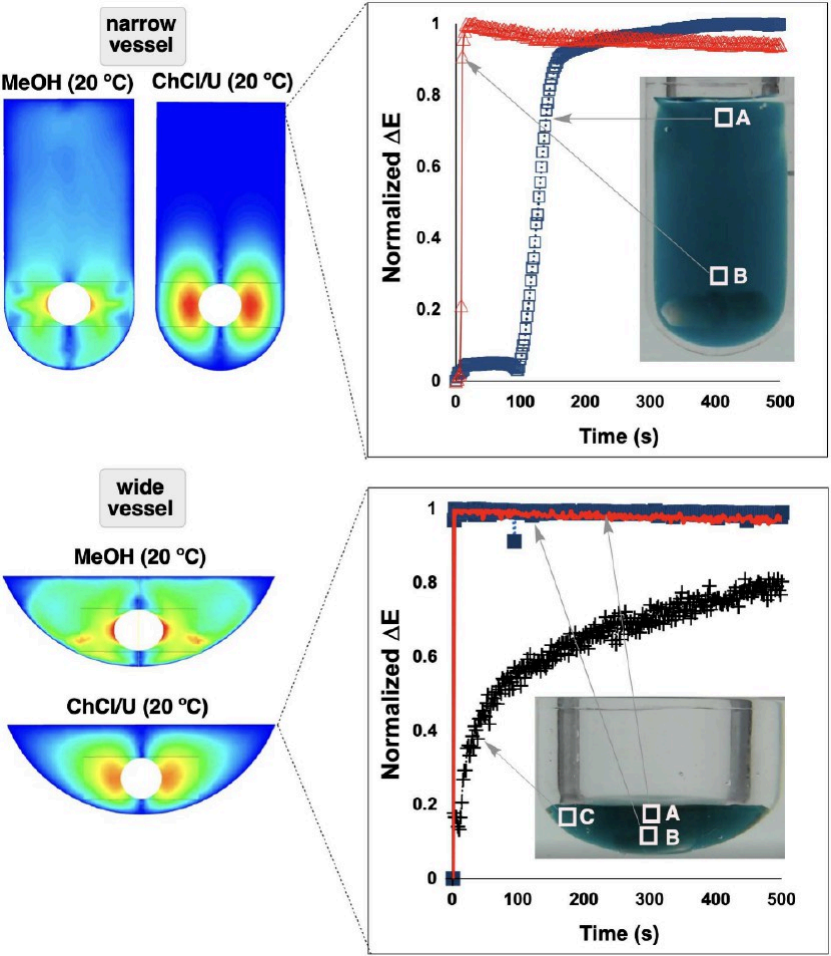
Computer vision validation of CFD mixing simulations. **Top:** In a narrow vessel, CFD velocity fields (left) predict distinct flow patterns for MeOH and ChCl/U (choline chloride–urea DES) at 20 °C. Normalized Δ*E* profiles for the viscous DES (right) confirm significantly slower homogenization at the top of the vessel (blue squares, ROI A) versus the bottom of the vessel (red triangles, ROI B). **Bottom:** In a wide vessel geometry, CFD simulations again capture the restricted flow fields for ChCl/U. Spatially resolved Δ*E* traces from three ROIs (A–C) reveal location-dependent mixing rates, with regions distant from the impeller (C) exhibiting prolonged heterogeneity. Vessel geometry and solvent viscosity jointly govern mixing efficiency, with direct implications for process scale-up.

The results were instructive. At the macroscopic level, simulated predictions of overall mixing time agreed well with our camera-derived plateau times across a range of impeller speeds.^94^ However, the spatially resolved analysis revealed subtleties that bulk comparisons would have missed. Grid-based Δ*E* profiles showed that both baffled and unbaffled reactor geometries achieved rapid mixing near the impeller, but vortex regions exhibited substantially slower homogenization. CFD simulations captured these spatial gradients, lending confidence to their predictive value for reactor design. Importantly, we found that baffled reactors achieved comparable mixing rates at lower stirring speeds, an insight with direct implications for energy-efficient process design. Feed point sensitivity analysis further demonstrated that reagent addition location significantly influenced mesomixing dynamics, with our spatial metrics capturing these effects in a manner directly comparable to CFD-predicted velocity fields.

The extension of this approach to viscous media proved equally revealing. Deep eutectic solvents (DES), increasingly important as sustainable reaction media, present severe mixing challenges due to their high viscosity.^95^ Applying our computer vision toolkit to DES formulations of choline chloride with ethylene glycol, glycerol, and urea, we observed mixing completion times ranging from seconds (for conventional solvent benchmarks) to over 60 min (for the most viscous DES systems). Temperature elevation from 25 to 60 °C reduced these times by up to 10-fold through viscosity reduction, and CFD simulations validated these observations by revealing the severe flow field restriction imposed by narrow vessel geometries under high-viscosity conditions.^95^ These findings carry practical consequences: reactions in DES that appear well-mixed by cursory visual inspection may harbor persistent spatial heterogeneity detectable through spatially resolved metrics.

The integration of these approaches (spatial segmentation for Δ*E*, *Contact* analysis, and GLCM texture analysis) transformed the computer vision efforts embodied in *Kineticolor* from a simple color tracking tool into a comprehensive mixing analysis platform. By providing multiple complementary perspectives on the same mixing process, we enabled users to choose the most appropriate metric for their specific application while maintaining consistency across scales and reaction types.

Looking back, our mixing analysis work made sophisticated engineering concepts accessible to the broader chemistry community. Most importantly, it demonstrated that the boundaries between chemical and engineering perspectives need not be barriers, and can instead be bridges to deeper understanding across an organization.

A significant limitation of the current approach is that it requires optically transparent or translucent vessels. Large-scale industrial mixing is still largely performed in opaque reactors, where direct camera observation is not feasible. Bridging strategies include transparent scale-down models that replicate industrial geometries, CFD simulations validated by computer vision at lab scale and then extrapolated, and emerging optical access points such as sight glasses or endoscopic probes. Fully opaque systems remain a genuine frontier for the technique.

## 6 Lessons Learned: Practical Camera Guidance

From our team’s experience, supporting both academic and industrial chemists in adopting computer vision, several recurring lessons have crystallized, and a workflow to support decision making is shown in Figure 18.

**Figure 18:**
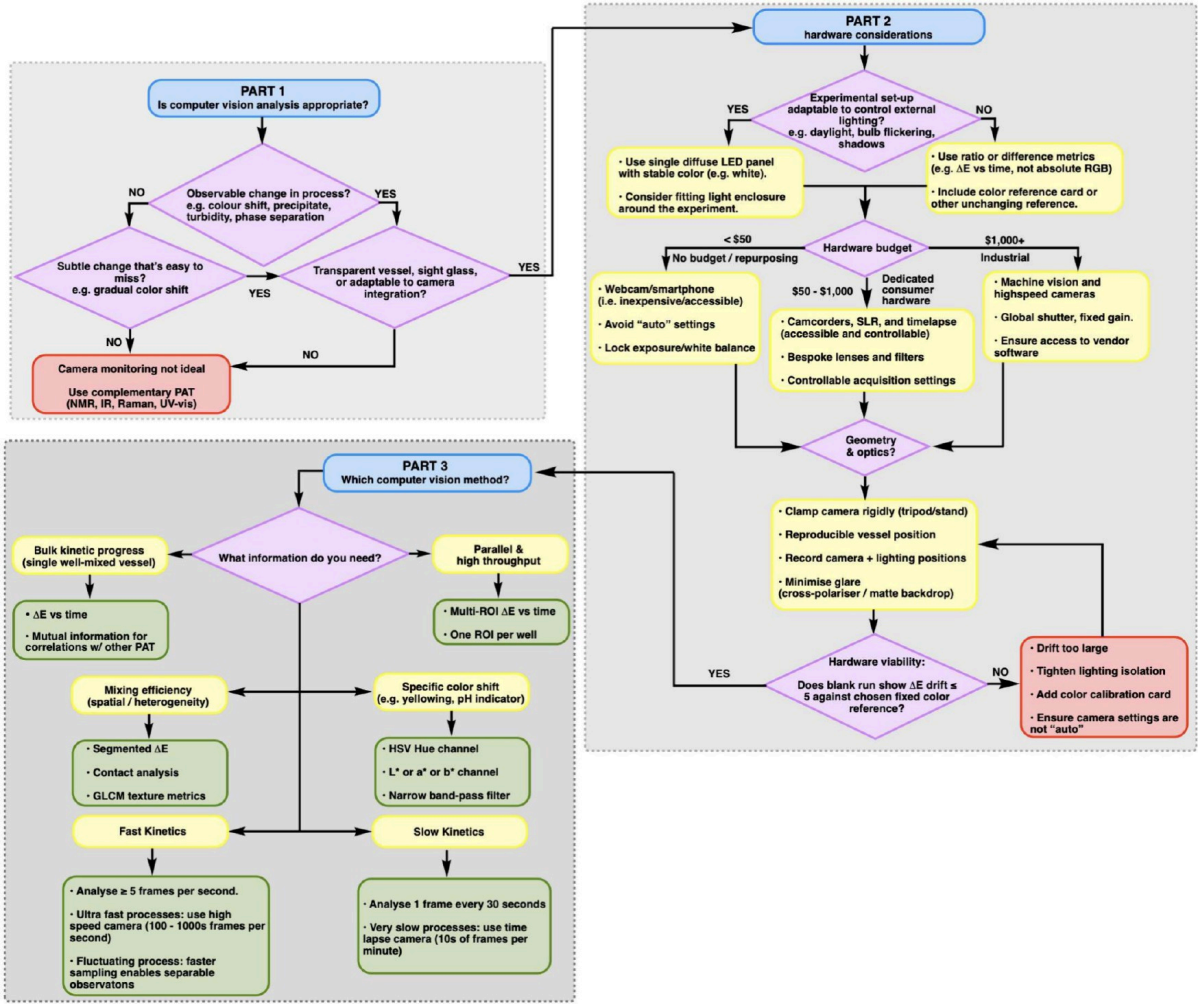
A decision tree to support strategic choice of computer vision setup for your monitoring application.

### 6.1 Stability and Hardware Democracy

The single most important lesson is deceptively simple: **stability trumps sophistication**. The most common failure mode we encounter is researchers attempting to capture hand-held footage, often with expensive cameras. Hand-held micromovements create apparent color changes (from shifting shadows and reflections) that can overwhelm the subtle chemistry being recorded. A $20 tripod or a spare clamp stand around the lab will improve results more than upgrading from a $200 to a $2000 camera.

Second, any camera that records video can generate useful chemical monitoring data. Whether using a smartphone, webcam, DSLR, security camera, or ATEX-rated camera for hazardous environments, access to the video file matters more than the camera specifications. We have successfully analyzed footage from devices ranging from decade-old webcams to cutting-edge industrial cameras with surprisingly little difference in the quality of chemical insights extracted. Even the simplest of webcams will collect a video with higher spatial and temporal resolution than can be acquired from most other reaction monitoring technologies. Only for the fastest reactions (on milli- and microsecond scales) and the most spatially sensitive processes do considerations of more sophisticated camera hardware become necessary.

### 6.2 Lighting and Data Management

Lighting stability often matters more than lighting intensity. Daylight shifts continuously throughout the day, creating systematic drift that can overshadow those chemical changes that play out over hours. We recommend compact diffuse LED photography boxes (typically under $100), which provide uniform, stable illumination ideal for computer vision. LED flat panels positioned behind reaction vessels enable transmitted light measurements with cleaner color data, and polarizing filters offer an elegant solution for managing glare from transparent glassware. All that said, if lab lighting is stable and not affected by outside environments, this can often suffice without further consideration.

Video data volumes can initially overwhelm new users: a 10 min reaction at 30 frames per second generates 18,000 measurements. Our software manages this through adjustable frame sampling rates and user-defined regions of interest. The greater interpretation challenge lies in the wealth of available color parameters (lightness, hue, saturation, Δ*E*, texture metrics) which creates a *paradox of choice*. We address this by encouraging users to correlate computer vision metrics with established analytical methods (e.g., Δ*E* against HPLC peak areas), building intuition through familiar validation.^4–6^ For most users, computer vision is about complementing existing data streams rather than replacing them.

Finally, if your camera of choice allows, switch from the “auto” to “manual” suite of settings; this primarily avoids issues around camera lens refocusing during a video recording, which can affect the level of light reaching the sensor and thus introduce nonchemical artifacts in your time series data.

### 6.3 Capabilities, Limitations, and Integration

Users often assume dramatic color changes are required, when in fact cameras detect far more subtle shifts than the human eye perceives (refer back to Table 2). To illustrate: two video frames that appear identical to a human observer can differ by Δ*E* ≈ 3–8, a range clearly resolvable by camera and well above the instrumental noise floor (Figure 19).^8^ We recommend that, even if by-eye you doubt there to be a significant visual signal present, attempt to measure it with a camera anyway.

**Figure 19:**
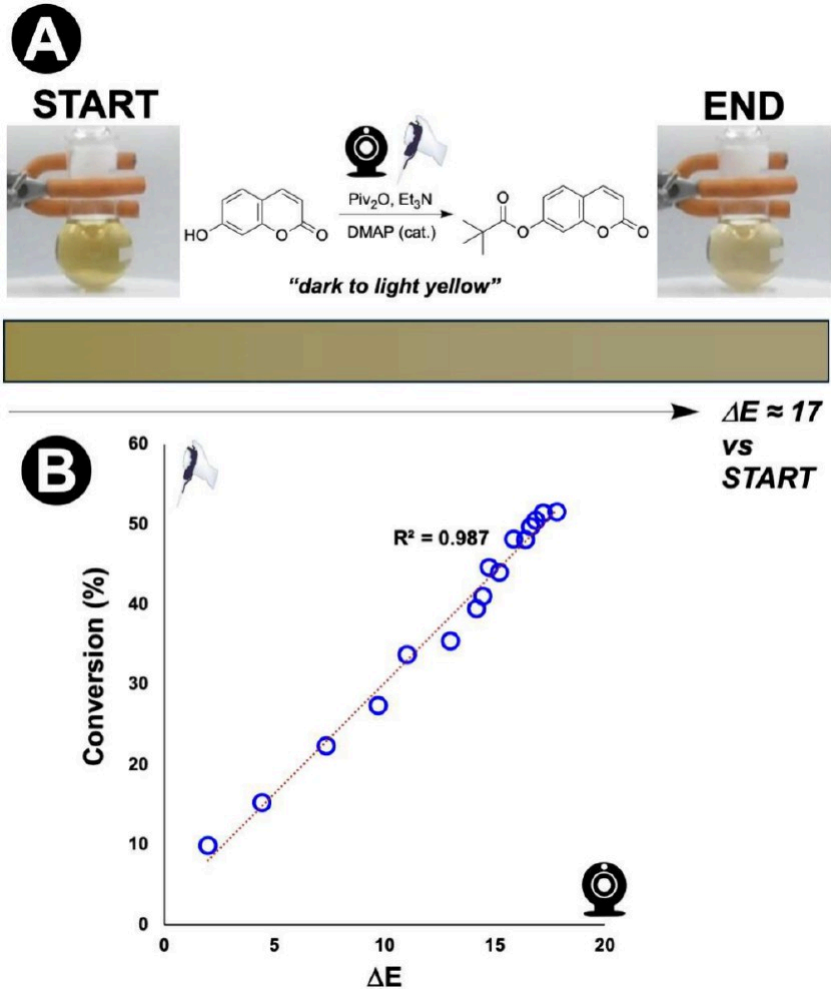
Quantifying a subtle color change by camera. **A:** Pivaloylation of umbelliferone produces a modest “dark to light yellow” shift (Δ*E* ≈ 17). **B:** Δ*E* correlates linearly with conversion (*R* ^2^ = 0.987), demonstrating that color changes easily dismissed by eye can yield reliable kinetic data when measured by a digital sensor (cf. Table 2).

For colorless-to-colorless reactions, where no intrinsic chromatic signal exists, the addition of a small quantity of a colorimetric or fluorescent indicator can render the process amenable to camera monitoring. pH-sensitive indicators (for acid- or base-generating reactions), redox-sensitive dyes, or solvatochromic probes can provide a visual proxy for reaction progress without perturbing the chemistry at low concentrations. This strategy effectively converts a “silent” reaction into one that a camera can track, extending the applicability of computer vision to systems that would otherwise be inaccessible.

Conversely, deeply opaque reaction mixtures present genuine challenges, as transmission-mode imaging is not feasible. In such cases, surface reflectance or backscatter geometries may still yield useful data: testing the angle of illumination relative to the camera detector can help, and optimizing setups for the camera to detect primarily surface reflection or scattering rather than transmission can have a meaningful impact on the signal obtained.^104^ However, when opacity precludes any meaningful color signal, alternative process analytical technology (PAT) tools such as Raman, infrared, or ultrasonic probes may be more appropriate, and we encourage users to consider computer vision as one component of a broader monitoring strategy rather than a universal solution.

The most important conceptual shift involves recognizing that computer vision provides bulk property information complementary to molecularly specific tools like NMR and FT-IR. For industrial implementations, integration with existing process analytical technology (PAT) infrastructure requires minimal modification, and the noninvasive nature of computer vision methods means camera addition does not disturb existing probe configurations or reactor geometries. The low cost and minimal infrastructure requirements lower the barrier to adoption, meaning computer vision can be evaluated with equipment most laboratories already possess.

## 7 Future Horizons

Several directions merit investigation. Future work should explore extracting the full complexity of a reaction not just from its color, but from the spatial structures of mixing and turbulence that a camera can reveal. A longer-term goal is to use a single video to infer rate constants, free energies, or mechanistic fingerprints that complement (and in some cases extend) the information accessible through probes, spectrometers, and single-point colorimeters alike (as discussed in the Introduction). A further aspiration is to treat every recorded experiment as a potential digital twin, replayable, reanalyzable, and reinterpretable as new algorithms emerge.

Several near-term advances are already within reach. The convergence of computer vision with self-driving laboratories represents perhaps the most immediate opportunity: autonomous platforms equipped with cameras would gain “eyes” capable of monitoring reaction progress, detecting anomalies, and triggering adaptive responses without human intervention.^44,45,105^ The time-resolved, multidimensional data sets generated by parallel computer vision monitoring are naturally suited to machine learning, offering rich training data for predictive models that could forecast reaction outcomes from early stage color trajectories alone. Beyond prediction, the spatiotemporal data sets captured by our methods could serve as permanent records of bulk reaction behavior, extending the value of a single experiment far beyond its initial purpose.

An under-explored but growing area for computer vision in process monitoring is solid-state reaction kinetics.^84,85,87^ Many solid-state transformations (polymorphic transitions, thermal decompositions) exhibit visible changes that are noted qualitatively but rarely quantified over time. Our peptide synthesis work, involving a solid–liquid multiphase system, provides partial precedent, and forthcoming studies on polyurethane condition monitoring and material swelling further demonstrate that if a transformation can be seen, a camera can extract a useful time series from it. The principal challenge remains that color information is restricted to surface layers, but this need not preclude meaningful kinetic insight.

Extending these methods to opaque industrial vessels remains an open challenge; transparent scale-down models and emerging optical access points (sight glasses, endoscopic probes) offer partial solutions.

On the measurement side, outstanding challenges remain. Our recent work on interdevice color correction has begun to address the reproducibility barrier posed by hardware variability between cameras.^55^ However, the fundamental gamut limitations we identified, where highly saturated colors exceed the sRGB color space and create artificial discontinuities in kinetic profiles, point to a deeper need for a standardized approach to interpreting color kinetic profiles relative to spectral imaging approaches. Using band-pass filters or (where budget allows) multispectral cameras could provide the spectral resolution necessary to deconvolve overlapping chromophores and extract molecularly richer information from video data. Finally, extending computer vision beyond transparent systems to reliably characterize opaque or heterogeneous reaction mixtures through surface texture analysis or multiangle illumination remains an open frontier that would substantially broaden the technique’s applicability.

Regardless of which of these questions excites you into action the most, we can collectively act (instead of rest) assured that computer vision for chemical process monitoring will be a rich vein of research for many years to come.

## Notes

Dedicated to the memory of Joseph McLachlan, whose love of lecturing and photography inspired our story.
